# The genetic architecture of structural left–right asymmetry of the human brain

**DOI:** 10.1038/s41562-021-01069-w

**Published:** 2021-03-15

**Authors:** Zhiqiang Sha, Dick Schijven, Amaia Carrion-Castillo, Marc Joliot, Bernard Mazoyer, Simon E. Fisher, Fabrice Crivello, Clyde Francks

**Affiliations:** 1 Language and Genetics Department, Max Planck Institute for Psycholinguistics, Nijmegen, the Netherlands; 2 Groupe d’Imagerie Neurofonctionnelle, Institut des Maladies Neurodégénératives, Centre National de la Recherche Scientifique, Commissariat à l’Energie Atomique, et Université de Bordeaux, Bordeaux, France; 3 Donders Institute for Brain, Cognition and Behaviour, Radboud University, Nijmegen, the Netherlands

**Keywords:** Genome-wide association studies, Magnetic resonance imaging, Behavioural genetics, Genetics of the nervous system, Psychiatric disorders

## Abstract

Left–right hemispheric asymmetry is an important aspect of healthy brain organization for many functions including language, and it can be altered in cognitive and psychiatric disorders. No mechanism has yet been identified for establishing the human brain’s left–right axis. We performed multivariate genome-wide association scanning of cortical regional surface area and thickness asymmetries, and subcortical volume asymmetries, using data from 32,256 participants from the UK Biobank. There were 21 significant loci associated with different aspects of brain asymmetry, with functional enrichment involving microtubule-related genes and embryonic brain expression. These findings are consistent with a known role of the cytoskeleton in left–right axis determination in other organs of invertebrates and frogs. Genetic variants associated with brain asymmetry overlapped with those associated with autism, educational attainment and schizophrenia. Comparably large datasets will likely be required in future studies, to replicate and further clarify the associations of microtubule-related genes with variation in brain asymmetry, behavioural and psychiatric traits.

## Abstract (web summary)

Genes associated with brain asymmetry are involved with the internal skeletons of cells, and are especially active in the embryo. Some of these genes are associated with handedness and risk for psychiatric disorders.

## Main

The human brain is characterized by various population-level asymmetries on its left–right axis^1^, including an overall ‘torque’ whereby the left hemisphere extends posteriorly and ventrally relative to the right, a left–right difference in frontal–occipital gradients of cortical thickness^2^, and hemispheric differences of morphology around the Sylvian fissure^3^. Many brain functions are also lateralized, including hand motor control and language which show left-hemisphere dominance in roughly 85% of people^4–13^. Altered brain or behavioural asymmetries have been reported in various cognitive and psychiatric disorders^7,14–17^, which suggests that population-typical asymmetries are linked to optimal human brain function.

Behavioural and anatomical brain asymmetries are already apparent in utero^1,18–20^, which indicates an early genetic-developmental programme of brain left–right axis formation^21,22^. Studies of visceral organ development (the heart, stomach, liver, etc.) have revealed that the generation of population-level asymmetry requires at least three important steps in the early embryo^23,24^: (1) the breaking of bilateral symmetry to create a left–right axis in a consistent orientation relative to the anterior–posterior and dorsal–ventral axes, (2) the triggering of different patterns of gene expression on the left and right sides of early embryonic structures and (3) the translation of asymmetric gene expression into lateralized morphology and organ placement.

In principle, establishing an embryonic left–right axis requires chirality at some level, that is, key biomolecules or cellular structures that exist in only one of two possible mirror forms. Life on Earth is based on L-form amino acids rather than the mirror D-form, and this chirality carries through to the macrostructure and movement of primary cilia^25,26^, which help to create the left–right axis of the visceral organs in embryos^25^. However, hemispheric dominances for language and hand motor control do not typically reverse in people with situs inversus of the viscera, that is, reversal of the visceral organs on the left–right axis, when caused by mutations in genes that encode primary ciliary components^27–30^. This observation indicates that there are distinct and possibly organ-intrinsic mechanisms at play in brain development, but such mechanisms remain unidentified. Therefore the genetic origins of human brain asymmetry remain unknown.

Post mortem studies that contrasted gene expression between the left and right sides of the embryonic central nervous system have identified molecular pathways that may be involved, including that the two sides may transition through developmental stages slightly out of synchrony with each other^31–33^. However, these studies were necessarily based on data from just handfuls of samples, because of limited availability arising from appropriate ethical, legal and practical concerns. An alternative approach to identify genetic influences on brain asymmetry is to relate genomic variation in large population datasets to variation in adult brain asymmetry. Only three loci have previously been reported at a genome-wide significant level to affect variation in adult human brain asymmetries, in studies targeted at single features of temporal lobe anatomy, and they did not yield broader insights into biological pathways^34,35^. Here we made use of the unprecedented sample size available through the 2020 release of the UK Biobank magnetic resonance imaging (MRI) data, in combination with genome-wide genotype data, to perform the first multivariate, brain-wide genetic analysis of human brain anatomical asymmetry. The results from this analysis also allowed us to test whether genetic polymorphisms that are associated with variation in brain asymmetry are also associated with neurodevelopmental disorders or other behavioural and psychological traits, using publicly available genome-wide association scan (GWAS) summary statistics for these traits.

## Results

### Heritabilities and genetic correlations of brain regional asymmetry measures

For each of 32,256 participants with post-quality-control MRI and genetic data (Methods), and each of 73 bilaterally paired regional measures of brain structure, we calculated hemispheric asymmetry indexes (AI) as (left − right)/((left + right)/2) (for 33 cortical surface area AIs, 33 cortical thickness AIs and 7 subcortical volume AIs; Supplementary Table 1). The measures were derived from cortical parcellation and subcortical segmentation of structural brain images (Methods). All but one of the regional mean AIs were significantly different from zero, indicating population-level asymmetries (Bonferroni-corrected *P* < 0.05, Supplementary Fig. 1 and Supplementary Table 2), consistent with previous reports^5,6^. For example, some language-related regions showed greater average left than right surface areas, including superior temporal and supramarginal cortex, and pars opercularis.

**Fig. 1:**
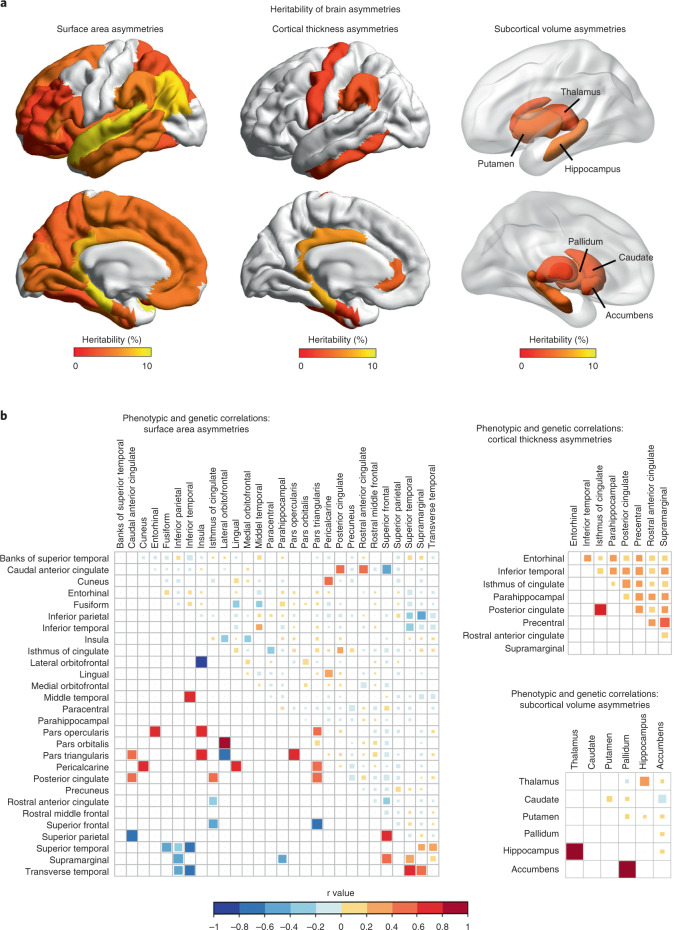
SNP-based heritability and correlation analysis of regional brain asymmetry measures. **a**, SNP-based heritability estimates for brain asymmetry measures. Only regions for which AIs were significantly heritable are indicated in colour. **b**, Genetic and phenotypic correlations between AIs. Phenotypic (upper right triangle) and genetic (lower left triangle) correlations between each pair of AIs. Only significantly heritable AIs that also have at least one significant phenotypic or genetic correlation after FDR correction are shown. The colours of the squares indicate the correlation coefficients according to the colour key, and their areas are proportional to the correlation coefficients.

GCTA^36^ software was used to estimate the single-nucleotide polymorphism (SNP)-based heritability (*h*^2^) for each AI, that is, the extent to which variance in each AI was linked to common genetic variation over the entire genome (Methods). Forty-two AIs showed significant SNP-based heritabilities (false discovery rate (FDR)-corrected *P* < 0.05), that is, 28 of the surface area AIs, 8 cortical thickness AIs and 6 subcortical volume AIs (Fig. 1a and Supplementary Table 3), ranging from 2.2% for the AI of entorhinal cortical thickness to 9.4% for the AI of superior temporal surface area. The overall pattern was consistent with previous, twin-based heritability analyses^5,6^.

SNP-based genetic correlation analysis (again using GCTA software) indicated overlapping genetic contributions to some of the AIs (Fig. 1b, Supplementary Fig. 2 and Supplementary Tables 4–10). Within a small number of cortical regions (Supplementary Table 10), surface area and thickness AIs had negative genetic correlations, which indicates that variants can have antagonistic effects on surface and thickness asymmetries of these regions.

### Multivariate genome-wide association analysis

**Fig. 2:**
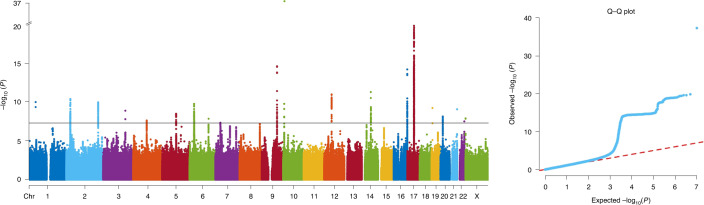
Multivariate GWAS analysis of regional brain asymmetries in 32,256 participants. Manhattan plot for multivariate GWAS across asymmetries of surface area, cortical thickness and subcortical volumes. The red dashed line indicates the significance threshold *P* < 5 × 10^−8^ (Methods). The Q–Q plot is also shown.

We performed a multivariate (mv)GWAS for 9,803,522 SNPs, using meta-canonical correlation analysis as implemented in MetaPhat^37^, with the 42 AIs that had significant SNP-based heritability. This analysis tested each SNP separately for its simultaneous associations with all 42 AIs (Methods). A multivariate approach had the dual advantages of achieving data reduction and increasing statistical power compared with running 42 separate univariate GWAS. FUMA^38^ software was used to clump mvGWAS results on the basis of linkage disequilibrium (LD), and identify lead SNPs (maximally associated SNPs) at each associated locus. There were 21 distinct genomic loci at the 5 × 10^−8^ significance level associated with different aspects of brain asymmetry (Fig. 2, Table 1 and Supplementary Fig. 3), represented by 27 independent lead SNPs (with pairwise LD *r*^2^ < 0.1, Table 1).

**Table 1:** Genomic loci associated with brain asymmetries on multivariate analysis. All lead SNPs are shown

Genomic locus	Lead SNP	Position	Functional category	Effect allele	Effect allele frequency	mvGWAS *r* value	mvGWAS *P* value	Nearest gene	Central asymmetry indexes^a^
1	rs6658111	1p33	Intergenic	G	0.37	0.0631	9.75 × 10^−11^	AL356458.1	Parahippocampal (SA), superior frontal (SA), parahippocampal (CT)
2	rs62130503	2p23.3	NcRNA_intronic^b^	T	0.05	0.0630	1.22 × 10^−10^	*MAPRE3*	Thalamus (SUB), parahippocampal (SA)
2	rs12617392	2p23.3	Intronic	A	0.44	0.0638	4.02 × 10^−11^	*CGREF1*	Inferior temporal (SA), caudal anterior cingulate (SA), isthmus of cingulate (SA)
3	rs368536282^c^	2q34	Intergenic	T	0.03	0.0631	1.07 × 10^−10^	*MAP2*	Superior frontal (SA), accumbens (SUB), posterior cingulate (CT)
4	rs2279829	3q24	3′ UTR^d^	T	0.22	0.0613	1.26 × 10^−9^	*ZIC4*	Isthmus of cingulate (CT), precuneus (SA), posterior cingulate (CT), fusiform (SA)
5	rs9307052^c^	4q22.1	Intronic	T	0.11	0.0591	2.27 × 10^−8^	*FAM13A*	Rostral anterior cingulate (CT), posterior cingulate (CT), medial orbitofrontal (SA)
6	rs869219775	5q15	Intergenic	T	0.14	0.0606	3.06 × 10^−9^	*NR2F1*	Inferior parietal (SA), transverse temporal (SA)
7	rs7781	6p21.33	Downstream	G	0.24	0.0628	1.62 × 10^−10^	*TUBB*	Isthmus of cingulate (CT), rostral anterior cingulate (CT), pars triangularis (SA)
8	rs9385385	6q22.31	ncRNA_intronic^b^	T	0.45	0.0595	1.37 × 10^−8^	*NCOA7*	Posterior cingulate (CT), pericalcarine (SA)
9	rs6947352	7p14.3	Intronic	A	0.31	0.0585	4.38 × 10^−8^	*BBS9*	Banks of the superior temporal sulcus (SA)
10	rs911934	9q22.33	Intergenic	G	0.70	0.0699	2.39 × 10^−15^	*GALNT12*	Inferior parietal (SA), isthmus of cingulate (SA), precuneus (SA), paracentral (SA), supramarginal (SA), entorhinal (CT)
11	rs41298373	10p14	Exonic	A	0.10	0.0940	4.75 × 10^−38^	*ITIH5*	Superior temporal (SA), parahippocampal (SA), fusiform (SA), inferior temporal (CT), transverse temporal (SA)
12	rs10783306^c^	12q13.12	Intergenic	C	0.33	0.0647	9.99 × 10^−12^	*TUBA1B*	Superior frontal (SA), entorhinal (SA), medial orbitofrontal (SA), pars triangularis (SA)
13	rs160459	14q23.1	Intergenic	C	0.46	0.0652	4.98 × 10^−12^	*DACT1*	Banks of the superior temporal sulcus (SA), transverse temporal (SA), pericalcarine (SA)
14	rs201816193	14q23.1	Intergenic	G	0.12	0.0621	4.38 × 10^−10^	*DAAM1*	Isthmus of cingulate (SA), cuneus (SA)
15	rs72813426	16q24.3	Intronic	G	0.24	0.0685	2.45 × 10^−14^	*SPIRE2*	Paracentral (SA), isthmus of cingulate (SA), middle temporal (SA)
15	rs111398992^c^	16q24.3	Intronic	T	0.13	0.0694	5.99 × 10^−15^	*TUBB3*	Isthmus of cingulate (CT), fusiform (SA), rostral anterior cingulate (CT), pericalcarine (SA)
16	rs55938136	17q21.31	NcRNA_intronic^b^	G	0.22	0.0695	4.91 × 10^−15^	*CRHR1*	Parahippocampal (SA), middle temporal (SA), pallidum (SUB), hippocampus (SUB), pars triangularis (SA)
16	rs35908989	17q21.31	Intronic	C	0.23	0.0595	1.34 × 10^−8^	*MAPT*	Supramarginal (SA), caudate (SUB)
16	rs35853889	17q21.31	3′ UTR^d^	TG	0.19	0.0765	1.43 × 10^−20^	*MAPT*	Rostral anterior cingulate (CT), cuneus (SA), isthmus of cingulate (SA), parahippocampal (SA), rostral anterior cingulate (SA), parahippocampal (CT)
16	rs80103986^c^	17q21.31	Intronic	T	0.20	0.0708	5.16 × 10^−16^	*KANSL1*	Parahippocampal (SA), middle temporal (SA), pallidum (SUB), hippocampus (SUB), pars triangularis (SA)
16	rs568039055	17q21.31	3′ UTR^d^	C	0.20	0.0692	7.87 × 10^−15^	*LRRC37A2* *ARL17A*	Parahippocampal (SA), isthmus of cingulate (SA), rostral anterior cingulate (CT), cuneus (SA)
17	rs11672092^c^	19p13.3	Intronic	T	0.22	0.0619	5.69 × 10^−10^	*TUBB4A*	Isthmus of cingulate (CT), lateral orbitofrontal (SA), middle temporal (SA)
18	rs6135555	20p12.1	Intronic	A	0.39	0.0600	7.00 × 10^−9^	*MACROD2*	Pericalcarine (SA), caudate (SUB)
19	rs7283026	21q22.3	Intronic	C	0.27	0.0616	8.42 × 10^−10^	*COL18A1*	Supramarginal (SA), transverse temporal (SA)
20	rs9615351	22q13.31	Exonic	G	0.25	0.0588	3.02 × 10^−8^	*CELSR1*	Isthmus of cingulate (CT), transverse temporal (SA)
21	rs12400461	Xp22.33	Intergenic	C	0.58	0.0595	1.24 × 10^−8^	*ASS1P4*	Inferior temporal (SA), pars opercularis (SA)

^a^Central traits for each SNP are those asymmetry indexes that contribute to its multivariate association (Methods). SA, surface area; CT, cortical thickness; SUB, subcortical volume.

^b^Intronic to a gene for a non-coding RNA.

^c^Lead variants are in high LD with handedness-associated variants.

^d^Untranslated region.

**Fig. 3:**
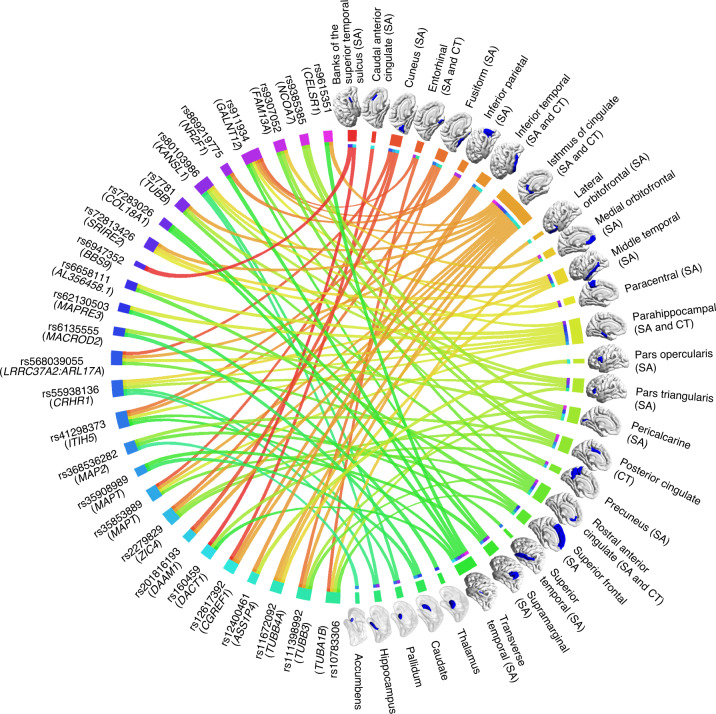
Overview of 27 independent lead variants associated with different regional brain asymmetries. Circle plot illustrating the 27 lead variants from mvGWAS (left) in relation to the central asymmetry indexes (right) underlying their specific multivariate associations. Different colours indicate different lead variants or regional asymmetries. Lines linking lead variants to regional asymmetries are coloured according to the regions. The closest genes to the lead variants are shown. Most central asymmetry indexes are of regional surface areas, and some variants affected multiple asymmetries of different types. SA, surface area; CT, cortical thickness; SUB, subcortical volume.

For each lead SNP, phenotype decomposition^37^ identified the ‘central’ AIs that contributed to its multivariate association (Supplementary Table 11). Most central AIs associated with the 27 lead SNPs were distributed in core regions of the language system (for example, lateral temporal, pars opercularis and supramarginal) or limbic system (for example, cingulate, orbitofrontal and mesial temporal cortex; Fig. 3). For example, the most significant SNP, with multivariate *r* = 0.094, *P* = 4.75 × 10^−38^ (rs41298373 on 10p14) had five central AIs: the minor allele was associated with a leftward shift of surface area asymmetry for two lateral temporal regions, a rightward shift of surface area asymmetry for two medial temporal regions and a leftward shift of cortical thickness asymmetry in the inferior temporal gyrus (Supplementary Table 11). All lead SNPs were associated with at least one cortical regional surface area AI as one of their central traits. Thirteen lead SNPs were associated with cortical thickness AIs, and five lead SNPs were associated with subcortical volume AIs. Two lead SNPs, rs35853889 and rs6658111, were associated with the AIs of both surface area and cortical thickness within the same region, that is, parahippcampal area and thickness AIs were associated with both of these SNPs, and rostral anterior cingulate area and thickness AIs were associated with rs35853889. In addition, five lead SNPs affecting cortical surface area AIs were also associated with AIs of subcortical volumes (Table 1 and Fig. 3), and the locus on 17q21 was associated with asymmetries of cortical surface area, thickness and subcortical volume (Table 1). The univariate associations of lead variants separately with left and right hemispheric measures are presented in Supplementary Table 11.

### Functional annotations of genomic loci associated with brain asymmetry

FUMA^38^ software applied three strategies to annotate candidate SNPs to genes at significantly associated loci (Methods): physical position, expression quantitative trait locus (eQTL) information and chromatin interactions (Supplementary Table 12 and Supplementary Figs. 4 and 5). Here we summarize and cite notable information on each of the lead SNPs:

Ten of the loci had annotations involving cytoskeleton-related genes: On 1p33, rs6658111 is close to AL356458.1, a pseudogene of *MTMR14* (myotubularin related protein 14). On 2p23.3, rs62130503 is intronic to *MAPRE3* (microtubule associated protein RP/EB family member 3a), and rs12617392 is a brain eQTL^39^ of *MAPRE3*. Located between these two SNPs on 2p23.3 is also *AGBL5*, which is a post-translational modifier of tubulin^40,41^. On 2q34, rs368536282 is close to *MAP2* (microtubule associated protein 2), a well-known dendrite-specific marker of neurons^42^, previously implicated in left-handedness by GWAS analysis^43–45^. On 6p21.33, rs7781 is in the 3′ untranslated region (UTR) of *TUBB* (tubulin beta class I). On 12q13.12, rs10783306 is close to the alpha tubulin gene *TUBA1B*. This variant is also in high LD with a handedness-associated variant, rs11168884 (*r*^2^ = 0.89)^44^. On 14q23.1, two lead variants for two independent genomic loci, rs160459 and rs201816193, showed evidence for cross-locus chromatin interaction via the promoters of nearby genes in foetal cortex^46^ (Supplementary Fig. 4). The former is near to *DACT1*, a locus which has been reported to associate with superior temporal sulcus depth^34^, while the latter is close to *DAAM1*, which modulates the reorganization of the actin cytoskeleton and the stabilization of microtubules^47,48^. Two lead variants on 16q24.3, rs72813426 and rs111398992, are in introns of *SPIRE2* and the tubulin gene *TUBB3*, respectively, both of which are key proteins in cytoskeleton organization^49,50^. rs111398992 is also in high LD with a handedness-associated variant, rs4550447 (*r*^2^ = 0.94)^44^. On 17q21.31 there were five independent lead SNPs: rs35908989 is intronic to *MAPT* which encodes microtubule-associated protein tau, and rs55938136, rs35853889 and rs568039055 are brain eQTLs^39,51,52^ of *MAPT*, while rs80103986 is in high LD (*r*^2^ = 0.91) with handedness-associated variant rs55974014^44^. On 19p13.3, rs11672092 is intronic to the tubulin gene *TUBB4A*, and in high LD with rs66479618 (*r*^2^ = 0.88), another handedness-associated SNP^44^.

The 11 other loci did not have obvious microtubule-related annotations, but most had annotations related to brain phenotypes or development: On 3q24, rs2279829 is a cortical eQTL^39^ of *ZIC4*, which is involved in visual and auditory pathway development^53^. rs9307052 on 4q22.1 is in high LD (*r*^2^ = 0.99) with the handedness-associated variant rs28658282^44^. On 5q15, rs869219775 is close to *NR2F1*, which is involved in neural activity during cortical patterning^54^. On 6q22.31-q22.32, rs9385385 is close to *NCOA7*, a nuclear receptor co-activator with its most abundant expression in the brain^55^. On 7p14.3, rs6947352 is intronic to *BBS9*, which causes Bardet–Biedl syndrome when mutated, involving retinopathy and intellectual disability^56,57^. On 9q22.33, rs911934 is located in a region having a chromatin interaction with *TRIM14* in adult cortex^46^ (Supplementary Fig. 4), a gene which may activate Wnt/β-catenin signalling and affects mesodermal versus ectodermal differentiation of embryonic stem cells^58^. On 10p14, rs41298373 is a predicted deleterious missense coding variant in *ITIH5*, which was previously reported to affect planum temporale volumetric asymmetry^35^. Inter-alpha-trypsin inhibitor proteins are involved in extracellular matrix stabilization^59^. On 20p12.1, rs6135555 is in a region having a chromatin interaction with the *FLRT3* promoter in neural progenitor cells^60^ (Supplementary Fig. 4), a gene which regulates axon guidance and excitatory synapse development^61^. On 21q22.3, rs7283026 is intronic to *COL18A1*, involved in neural tube closure and mutated in Knobloch syndrome^62^, which can include skull abnormalities. On 22q13.31, rs9615351 is an exonic variant of a gene involved in planar cell polarity, *CELSR1*^63^. On Xp22.33, rs12400461 is close to pseudogene *ASS1P4* and upstream of *MXRA5*; the latter encodes a matrix remodelling-associated protein and is implicated in autism^64^.

**Fig. 4:**
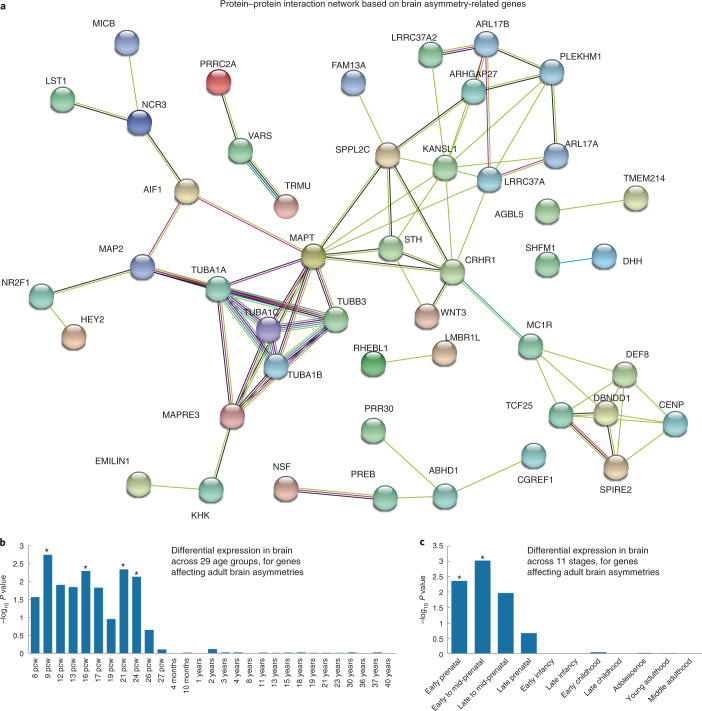
Functional annotations of variants associated with brain asymmetry. **a**, Brain asymmetry-associated genes integrated into a protein–protein interaction network. Proteins are represented by nodes. Edges between nodes represent different types of protein–protein interactions according to the STRING database (Methods), including known interactions (turquoise and dark purple represent interactions identified by curated databases and biological experiments, respectively), predicted interactions (green, red and blue represent interactions predicted by gene neighbourhood, gene fusions and gene co-occurrence, respectively) and others (yellow, black and light purple represent interactions determined by text mining, co-expression and protein homology, respectively). Coloured nodes represent the queried proteins. Only medium-confidence (>0.4) links were retained, and disconnected proteins are not shown. **b**, Relation between gene-based association with brain asymmetries and relatively higher mRNA expression in the human brain at particular ages, using BrainSpan data from 29 age groups. Asterisks indicate significant age groups meeting *P* < 0.05 with FDR correction. pcw, post-conceptional weeks. **c**, Relation between gene-based association with brain asymmetries and relatively higher mRNA expression in the human brain at particular ages, using BrainSpan data from 11 defined age groups. Asterisks indicate significant groups meeting *P* < 0.05 with FDR correction.

To further link asymmetry-associated SNPs to genes, the MAGMA^65^ software was used to perform genome-wide gene-based association analysis^65^ based on the results from mvGWAS. There were 57 significant genes at Bonferroni-corrected *P* < 0.05 (Supplementary Fig. 6 and Supplementary Table 13). Five of these genes were previously associated with handedness^44^: *MAP2*, *FAM13A*, *TUBA1B*, *TUBB3* and *CRHR1*. Forty-three of the 57 genes have been reported to associate with educational attainment^66^ and 15 with intelligence^67^ (Supplementary Table 14). For the proteins encoded by the 57 genes, there were 80 known or putative pairwise interactions in the STRING database^68^, compared with 8 interactions expected for a random set of this size from the whole proteome (*P* < 1 × 10^−16^). This observation supports the validity of our mvGWAS association findings, as random noise would not lead to such functional clustering. Microtubule-related genes (for example, *MAP2*, *MAPT*, *SPIRE2* and *TUBA1A*) linked different clusters together in the largest protein interaction network (Fig. 4a).

We also used the genome-wide, gene-based *P* values for functional enrichment analysis using MAGMA^65^, in relation to 7,343 Gene Ontology ‘biological process’ sets defined within the MSigDB^69^ database. The gene set ‘regulation_of_microtubule_binding’ (*P* = 3.73 × 10^−6^) showed significant enrichment (adjusted *P* < 0.05, Bonferroni correction, Supplementary Table 15). Significant enrichment within various microtubule-related sets, such as ‘microtubule_cytoskeleton_organization’ (*P* = 2.19 × 10^−7^) and ‘microtubule_based_process’ (*P* = 2.36 × 10^−6^), was also found when using the list of single closest genes (Table 1) to the 27 lead SNPs (Supplementary Table 16). Enrichment in microtubule-related sets was not reported in a recent GWAS of bilaterally averaged cortical surface area and thickness measures in 51,665 individuals^70^, suggesting a particular involvement in hemispheric asymmetry rather than bilateral measures. We observed no statistically significant relation of our gene-based association *P* values with differential expression across cell types (Methods).

Testing our genome-wide, gene-based *P* values with respect to human gene expression data from the BrainSpan^71^ database, from either 29 age groups or 11 defined developmental stages, we found relatively higher mRNA expression of brain-asymmetry-associated genes during early-prenatal (*P* = 4.27 × 10^−3^) and early–mid-prenatal (*P* = 9.37 × 10^−4^) stages, from 9 (*P* = 1.84 × 10^−3^) to 24 (*P* = 7.36 × 10^−3^) post-conceptional weeks (FDR-corrected *P* values <0.05) (Fig. 4b,c and Supplementary Table 17). This is consistent with the fact that various anatomical asymmetries of the brain are already visible in utero^18,19^, and supports the existence of an early developmental mechanism for establishing the brain’s left–right axis^31,32,72^.

### Genetic overlap of brain asymmetry with other traits

**Fig. 5:**
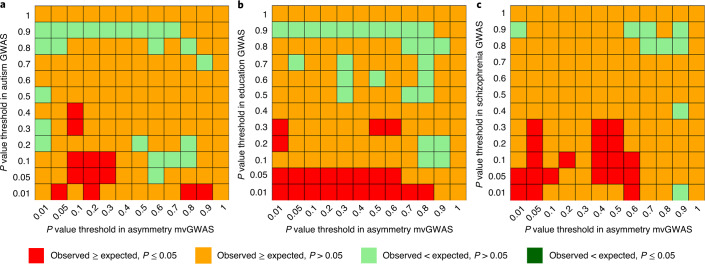
Genetic overlaps between brain asymmetries and other traits. **a**–**c**, Heatmap plots illustrating pleiotropic effects between brain asymmetries and autism (**a**), educational attainment (**b**) and schizophrenia (**c**), based on per-SNP GWAS *P* values for these traits from previous studies (Methods), in relation to the mvGWAS *P* values from the present study of brain asymmetries.

We next used iSECA software^73^ to perform genetic overlap analysis with our mvGWAS results in relation to GWAS summary statistics from neurodevelopmental disorders, behavioural and psychological traits which have been reported to associate phenotypically with aspects of structural and/or functional brain asymmetry: attention deficit hyperactivity disorder^16,74–77^, autism spectrum disorder^15,78–82^, educational attainment^66,83,84^, handedness^2,4,45^, intelligence^67,85–88^ and schizophrenia^17,89–93^. There was evidence for genetic overlap between brain asymmetries and autism (*P* = 0.005), educational attainment (*P* = 0.001) and schizophrenia (*P* = 0.002) which remained significant at Bonferroni-corrected *P* < 0.05 (Fig. 5, Supplementary Figs. 7 and 8 and Supplementary Table 18). In other words, SNPs that showed lower (more significant) association *P* values in our mvGWAS for brain asymmetry showed a statistically significant tendency to also show lower *P* values in previous, large-scale GWAS of autism, educational attainment and schizophrenia. Although we did not observe genetic overlap of brain asymmetry with handedness at a genome-wide level, we did note individual loci in common between these traits (above). In addition, we found no overlap between our mvGWAS results and those from a previous GWAS of intracranial volume in 32,438 participants^94^ (Supplementary Table 18, Supplementary Figs. 7 and 8), which again indicates that the genetic architecture of brain asymmetry is largely distinct from brain size.

### Validation of lead SNPs associated with brain asymmetry

To achieve a reasonable level of genetic homogeneity in our mvGWAS, we had excluded any individuals not annotated as having ‘white British ancestry’ (through a combination of self-report and clustering based on principal components that capture major axes of ancestral diversity from the genotype data^95^ (Methods)). The UK Biobank includes additional participants who self-identify as being ‘white’, but who did not self-identify as British, or did not cluster genetically with the bulk of the ‘white British’ ancestry participants (Methods). After applying the same quality control criteria to these additional participants as in our discovery mvGWAS (except with respect to ancestry), and imposing the extra criterion that relatedness kinship coefficients should not be greater than 0.0442 with any participants from the discovery mvGWAS, data were available from 3,600 participants for an independent replication set. We tested each of the 27 lead SNPs from the mvGWAS in the replication set, using the same approach as the mvGWAS analysis, except that 40 genetic principal components were used as covariates to control for the greater degree of ancestral diversity in the replication set.

Ten of the 27 independent lead SNPs from the discovery mvGWAS showed association *P* values < 0.05 on multivariate testing in the replication set (Supplementary Table 19). The combined *P* value of the remaining 17 lead SNPs was *P* = 3.3 × 10^−4^ in the replication set (calculated by Stouffer’s method), which we confirmed by permutation with respect to 10,000 repeat random samplings of 17 SNPs from the whole genome in the replication set (permutation-based *P* = 4 × 10^−4^). This indicates that the limited sample size of the replication set, compared with the discovery set, did not provide adequate power to replicate at the level of some individual SNPs, but that in combination there was evidence for replication. Moreover, among the 17 SNPs that showed *P* > 0.05 on multivariate testing in the replication set, some showed association *P* < 0.05 on univariate testing of the specific central traits identified for those SNPs in the discovery mvGWAS (Supplementary Table 19). It is also worth noting that 4 of these 17 SNPs (or SNPs in high linkage disequilibrium with them) have been reported to associate with left-handedness at a genome-wide significant level^44^ (see above for details), which is an additional form of validation with respect to a phenotype related to brain asymmetry. As also mentioned above, the high degree of functional clustering of genes identified through gene-based association testing is another form of support for the association results in the mvGWAS.

## Discussion

Despite the importance of asymmetry as an organizing feature of the human brain, the early developmental processes which establish its left–right axis are unknown. In this study, we carried out multivariate GWAS analysis which identified 21 genetic loci associated with different aspects of adult brain anatomical asymmetry. Functional annotation implicated genes particularly involved in microtubule organization and prenatal brain development. Our study therefore sheds new light on the molecular genetic foundations of human brain asymmetry. In addition, at the genome-wide level, SNPs associated with brain asymmetry overlapped with those associated with educational attainment, autism and schizophrenia, while five specific loci that are associated with both brain asymmetry and handedness were identified.

Previous studies in invertebrates and frog embryos have shown that the cytoskeleton plays a role in determining cellular chirality and asymmetrical patterning of other organs^96–102^. Cellular chirality refers to directional biases in cellular morphology, position, rotation or migration, which arise because of the inherent chirality of intracellular macromolecules such as those composing the cytoskeleton^96^, and also manifest in terms of the intracellular distributions of organelles^103^. For example, during early cell divisions in *Xenopus* (frog) embryos, the cytoskeleton has been reported to mediate asymmetric intracellular protein localization, as protein transport molecules move along cytoskeletal tracks within cells^104^. Thus the cytoskeleton can provide a directionally consistent, organ-intrinsic bias during embryonic development that acts as a determinant of morphological asymmetry^97,98,105^, arising from fundamental aspects of molecular and cellular biology.

As our study associated cytoskeletal and embryonically expressed genes with variation in adult human brain asymmetry, it is possible that these genes are involved in the establishment of left–right asymmetry during early brain development, through a mechanism involving cellular chirality. As mentioned in Main, at least some aspects of human brain asymmetry appear to be uncoupled from the developmental pathway that leads to left–right organization of the visceral organs, which involves cilia and the nodal pathway. A cytoskeletal-mediated mechanism of brain asymmetry may therefore be organ intrinsic^97,98,105^, that is, distinct from other pathways that establish broader aspects of body asymmetry.

In this study, we identified genetic loci that are associated with 42 heritable aspects of brain asymmetry through a multivariate, brain-wide approach. A multivariate approach can boost statistical power while achieving data reduction, compared with separate univariate analyses of individual brain traits^37^. A single set of genome-wide association results, pertaining simultaneously to multiple aspects of brain asymmetry, was then taken forward into functional annotation and downstream analyses, such as testing for genetic overlaps with other traits. The multivariate approach therefore helped to detect and interpret key aspects of the genetic architecture of brain asymmetry, without the noise inherent in repeat univariate testing. An important challenge remained to identify the particular brain traits that drove the multivariate associations at each locus, which was achieved in MetaPhat^37^ by decomposing associations into sets of ‘central’ traits on the basis of the Bayesian information criterion and canonical correlation *P* values.

A consequence of the multivariate approach is that it does not yield univariate association effect sizes, and therefore mvGWAS results cannot be used for standard genetic correlation analyses, such as is performed with LD score regression^106^. Therefore, we used iSECA^73^ to explore the genetic overlap of brain asymmetry with neurodevelopmental disorders, behavioural and psychological traits. This analysis was based on SNP-wise *P* values of association over the whole genome, that is, a genetic overlap was found when the SNPs tending to show low *P* values in our mvGWAS for brain asymmetry also tended to show low *P* values in publicly available GWAS summary statistics for a given disorder or trait. We found significant genetic overlaps of brain asymmetry with autism, schizophrenia and educational attainment, which suggests that genes affecting brain asymmetry also influence these traits. This is in line with literature that has shown phenotypic associations between altered brain asymmetry and these traits (see Main), and indicates that such phenotypic associations are contributed to an extent by genetic factors. As we found that brain asymmetry-related genes tend to be especially highly expressed in the embryonic brain, it seems likely that the genetic overlap of brain asymmetry and disorders reflects a genetic susceptibility to alterations of early neurodevelopment away from the typical trajectory. However, brain asymmetry continues to develop throughout the lifespan^107,108^, and the UK Biobank consists of middle- to older-age adults, so that our mvGWAS may have also identified genetic factors that affect brain asymmetrical changes later in life.

Further research, for example, using Mendelian randomization^109^, will be needed to understand whether brain asymmetries mediate gene–disorder associations in a causal sense, or whether altered brain asymmetry and disorder susceptibility are two distinct consequences arising from a partly overlapping genetic basis. It will also be important to map, on a brain-regional basis, which aspects of asymmetry show the strongest genetic overlaps with disorder susceptibility. Larger imaging–genetic datasets may be needed to support causal mediation and mapping studies with respect to disorders, as the present genetic association analysis was based on brain-wide asymmetry (albeit in a multivariate context).

Many of the brain asymmetries were strong and directional at the population level, but their heritabilities were generally low, ranging up to 10%. This suggests that developmental mechanisms for brain asymmetry are tightly constrained and largely genetically invariant in the population, and that environmental factors and/or developmental randomness are responsible for most variability^45,110–112^. A cytoskeleton-based origin of brain asymmetry would fit this scenario, as the cytoskeleton is essential for various fundamental functions in cellular biology, beyond axis formation^113,114^. Previous, twin- and family-based analyses^5,6^ have reported heritabilities up to roughly 25% for some of the same asymmetry measures that we analysed in the present study, with an overall similar regional pattern. Higher heritabilities were found particularly for regions that are important in the language system (for example, superior temporal cortex) and limbic system (for example, medial temporal and cingulate cortex). Twin-based heritability is often measured to be higher than SNP-based heritability, which may be expected because SNPs are just one class of genetic variation, and also because twin studies can overestimate heritability when certain assumptions are not fully met^115^. As twin studies have not indicated effects of shared environment on brain aymmetries^5,6^, early developmental randomness is likely to cause most variation^116^.

We did not correct for handedness as a covariate in our genetic analyses, as it is generally not advisable to correct for covariates which are themselves partly heritable. This is because biased genetic effects can be measured with respect to the target trait^117^ (in this case, brain asymmetry). Handedness cannot therefore be treated safely as a confound variable when analysing brain asymmetry. We were interested in any genetic effects associated with brain asymmetry, regardless of whether they might also be shared with other traits such as handedness. Having identified genetic variants associated with brain asymmetry, we then queried post hoc whether they have been reported as significant in previous GWAS of handedness in over 1 million people^44^. We did not observe a significant genetic overlap between structural brain asymmetry and handedness at the genome-wide level, which again may be due to the relatively low SNP-based heritabilities of these traits, in combination with limited statistical power in the present sample size for this kind of analysis. However, five individual SNPs associated with both brain asymmetry and handedness were identified, which suggests that a significant genome-wide overlap might be detected when using a larger dataset in the future.

The UK Biobank currently includes by far the largest single imaging–genetic dataset available to the scientific community. A limitation of the present study is the lack of a large, age-matched replication sample with comparable homogeneity of ancestry to the discovery mvGWAS analysis. We included an independent replication sample of 3,600 individuals from the UK Biobank, with greater diversity of ancestry than the 32,256 individuals of the primary mvGWAS. Association was replicated for 10 of the 27 independent lead SNPs from the mvGWAS, and for the remaining 17, their combined *P* value in the replication set was 3.3 × 10^−4^, indicating that a larger dataset would have supported replication at the level of more individual SNPs. Functional clustering of the closest genes to the 27 independent lead SNPs, according to microtubule-related biology and protein–protein interactions, also supported the validity of the mvGWAS findings, as this was unlikely to occur by chance. In addition, four of the SNPs that did not replicate individually at *P* < 0.05 (or SNPs in high linkage disequilibrium with them; see Results) were previously associated at a genome-wide significant level with left-handedness, which is an additional form of validation with respect to a behavioural asymmetry trait.

Another limitation of the present study is that functional enrichment analysis with respect to post-mortem, developmental gene expression was only possible from roughly 8 weeks post conception and onwards. The Brainspan database does not contain sufficient numbers of samples from earlier embryonic stages than this. It may be that the left–right brain axis is established extremely early in development, for example during formation of the neural tube, which begins in the third week post conception^118^. As noted in Main, possibilities for research using human embryos from this developmental stage are highly restricted. Gene expression studies using animal models may therefore be necessary to understand the establishment of the mammalian brain’s left–right axis.

In conclusion, our findings motivate genetic-developmental studies of left–right differentiation of the embryonic mammalian brain, focused on a possible cytoskeletal-mediated mechanism of axis formation. Our study also suggests that disruption of this mechanism may contribute to susceptibility to cognitive and psychiatric disorders, in line with asymmetry being an important aspect of healthy brain organization for many functions.

## Methods

### Participants

This study was conducted under UK Biobank application 16066, with C.F. as principal investigator. The UK Biobank is a general adult population cohort. The UK Biobank received ethical approval from the National Research Ethics Service Committee North West-Haydock (reference 11/NW/0382), and all of their procedures were performed in accordance with the World Medical Association guidelines. Informed consent was obtained for all participants. We used the brain imaging data released in February 2020, and data availability and processing (described below) resulted in a final sample of 32,256 participants of white British ancestry for the primary GWAS analysis, together with the structural MRI data and genotype data from the same participants. The age range of these participants was 45 to 81 years (mean 63.77 years), 15,288 were male and 16,968 were female. An independent replication dataset of 3,600 individuals was also drawn from the UK Biobank, who self-identified as white, but not British, or did not cluster genetically with the bulk of the ‘white British’ ancestry participants (see below). The age range of these participants was 45 to 80 years (mean 62.89 years), 1,574 were male and 2,026 were female.

### Genetic quality control

We downloaded imputed SNP genotype data from the UK Biobank data portal (bgen files; imputed data v3-release March 2018). We first excluded subjects with a mismatch of their self-reported and genetically inferred sex, with putative sex chromosome aneuploidies, or who were outliers on the basis of heterozygosity (principle-component-corrected heterozygosity >0.19) and genotype missingness (missing rate >0.05) as calculated by Bycroft et al.^95^. The primary analyses were restricted to participants with ‘white British ancestry’, which was defined by Bycroft et al. (‘in.white.British.ancestry.subset’)^95^, using a combination of self-report and cluster analysis on the basis of the first six principal components which capture genetic ancestry from the genome-wide genotype data. We randomly excluded one from each pair of remaining individuals who had a kinship coefficient >0.0442, as defined by Bycroft et al.^95^. Next, QCTOOL (v.2.0.6) and PLINK^119^ were used to perform genotype quality control: excluding SNPs with minor allele frequency <1%, Hardy–Weinberg equilibrium test *P* value <1 × 10^−7^ and imputation INFO score <0.7 (a measure of genotype imputation confidence). We also excluded multi-allelic SNPs because most of the downstream software (below) could not handle them. This resulted in 9,803,522 bi-allelic variants.

The same process was applied to derive the independent replication dataset of 3,600 individuals, except that these participants did not self-identify as British (although they did identify as ‘white’), or they did not fall within the bounds of the ‘white British ancestry’ cluster as defined by Bycroft et al.^95^. We also imposed the extra requirement that participants in the replication set should not show a kinship coefficient >0.0442 with any individual in the primary discovery dataset.

### Neuroimaging phenotypes and covariates

Brain anatomical measures of regional cortical surface area, cortical thickness and subcortical volumes were derived from the structural scans (Siemens Skyra 3-T MRI with 32-channel radiofrequency receive head coil) released by the UK Biobank Imaging Study (for the full protocol, see http://biobank.ndph.ox.ac.uk/showcase/refer.cgi?id=2367). Briefly, in vivo whole-brain T1-weighted MRI scans were used to perform cortical parcellation into 34 regions per hemisphere with the Desikan–Killiany atlas^120^, and 7 subcortical structural segmentations. Surface area was measured at the grey–white matter boundary, and thickness was measured as the average distance in a region between the white matter and pial surfaces. Details of the image quality control and processing are described elsewhere^121^. Given that the data for the temporal pole were reported as unreliable^121^, we only used 33 surface area, 33 cortical thickness and 7 subcortical volume measures in each hemisphere (Supplementary Table 1). Per measure, we removed data points greater than six standard deviations from the mean. Then, we calculated the AI for each matching pair of left and right measures, in each participant, as (left − right)/((left + right)/2). Given this definition, a positive AI reflects leftward asymmetry (greater left than right). The AI is a widely used measure in brain asymmetry studies^5,122,123^. The denominator ensures that the index does not simply scale with brain size, that is, the left–right difference is adjusted for the bilateral measure. For each AI, one-sample *t* testing was used to examine whether the population mean AI was significantly different from zero, with Bonferroni correction at 0.05 for multiple testing. Subsequently, the distributions of AIs were normalized by rank-based inverse normalization to minimize statistical artifacts. The normalized AIs were used as input for subsequent analysis.

The Desikan–Killiany atlas^120^ was derived from manual segmentations of sets of reference brain images. The labelling system incorporates hemisphere-specific information on sulcal and gyral geometry with spatial information regarding the locations of brain structures, and shows a high accuracy when compared with manual labelling results^120^. Accordingly, the mean regional asymmetries in the UK Biobank might partly reflect left–right differences present in the reference dataset used to construct the atlas. However, our study was focused primarily on comparing relative asymmetry between genotypes, at the regional level. The use of an asymmetrical atlas based on healthy individuals had the advantage that regional identification was likely to be accurate for structures that are asymmetrical in the general population, while taking hemisphere-specific information into account.

We also made use of continuous variables as covariates in heritability estimation and genome-wide association analysis (below), which were: age when attended assessment centre (UK Biobank fields 21003-2.0), nonlinear age, that is (age − mean_age)^2^, the first ten genetic principal components capturing population genetic diversity (fields 22009-0.1 to 22009-0.10) (or the first 40 principal components in the replication dataset with higher diversity of ancestry), scanner position parameters (*X*, *Y* and *Z* position: fields 25756-2.0, 25757-2.0 and 25758-2.0), T1 signal-to-noise ratio (field 25734-2.0) and T1 contrast-to-noise ratio (field 25735-2.0), plus categorical covariates which were: assessment centre (field 54-2.0), genotype measurement batch (field 22000-0.0) and sex (field 31-0.0).

### SNP-based heritability and genetic correlation analysis within th*e* UK Biobank data

From the primary dataset, 9,516,074 autosomal variants with minor allele frequencies >1%, INFO score >0.7 and Hardy–Weinberg equilibrium *P* > 1 × 10^−7^ were used to build a genetic relationship matrix using GCTA^36^ (version 1.93.0beta). Specifically for analyses using GCTA, we further excluded one random participant from each pair having a kinship coefficient higher than 0.025 based on the calculated genetic relationship matrix (as this analysis is especially sensitive to higher levels of relatedness), resulting in 30,315 participants. Genome-based restricted maximum likelihood (GREML)^36^ analyses were performed to estimate the SNP-based heritability for each AI, controlling for the above-mentioned covariates, and applying FDR 0.05 across the 73 AIs to define significantly heritable AIs. Bivariate GREML^124^ analysis was used to estimate genetic correlations between pairs of AIs, separately for cortical surface area, cortical thickness and subcortical volume AIs, with FDR correction at 0.05 for multiple testing.

### Multivariate genome-wide association analysis

In mvGWAS, a single association test is performed for each SNP in relation to multiple traits simultaneously. We used MetaPhat^37^ software to perform mvGWAS analysis across asymmetries for cortical surface area, cortical thickness and subcortical volume, including only the 42 AIs that had shown significant SNP-based heritability. MetaPhat performs meta-canonical correlation analysis, and uses univariate GWAS summary statistics as input from each separate AI, which were derived under an additive genetic model while controlling for the above-mentioned covariates, using BGENIE software (v1.2)^95^. Thus our mvGWAS tested effectively for association with 42 traits. This approach estimates the linear combination of traits that is maximally associated with genotype, which can differ for each SNP, while maintaining a correct false-positive rate. A total of 9,803,522 SNPs (see further above) were used for mvGWAS, spanning all autosomes and chromosome X. Statistically significant SNPs were considered as those with *P* < 5 × 10^−8^ in mvGWAS, which is a widely used threshold to account for multiple testing over the whole genome, in the context of LD in European-descent populations^125,126^.

MetaPhat also uses systematic criteria to define central traits which make the greatest contributions to significant multivariate associations, on the basis of an iterative process to optimize multivariate model properties with reference to canonical correlation analysis *P* values and the Bayesian information criterion^37^. For the lead SNPs at genome-wide significant loci (see below for how these were defined), we also performed post hoc analysis in which we examined their separate left and right hemispheric associations, using traits corresponding to the central AIs that were involved in the multivariate associations (Supplementary Table 11), again using BGENIE, an additive genetic model and covariates as described above.

As a sensitivity analysis, we re-ran the mvGWAS after excluding from the primary dataset 886 participants who had lifetime diagnoses of neurological conditions that could potentially disrupt brain structure (Supplementary Table 20). The significant mvGWAS loci were minimally affected by this exclusion (Supplementary Fig. 9).

### Identification of genomic risk loci and fun*c*tional annotations

FUMA (version v1.3.6)^38^, an online platform for functional annotation of GWAS results, was applied to the results from mvGWAS. A multi-step process, using default parameters, was used to identify distinct, significantly associated genomic loci, and independent lead SNPs within those loci. Briefly, on the basis of the pre-calculated LD structure from the 1000 Genomes European reference panel^127^, SNPs with genome-wide significant mvGWAS *P* values <5 × 10^−8^ that had LD *r*^2^ < 0.6 with any others were identified. For each of these SNPs, other SNPs that had *r*^2^ ≥ 0.6 with them were included for further annotation (see below), and independent lead SNPs were also defined among them as having low LD (*r*^2^ < 0.1) with any others. If LD blocks of significant SNPs are located within 250 kb of each other (default parameter), they are merged into one genomic locus. Therefore, some genomic loci could include one or more independent lead SNPs (Table 1). The major histocompatibility complex region on chromosome 6 was excluded from this process by default, because of its especially complex and long-range LD structure.

Functional annotations were applied by matching chromosome location, base-pair position, reference and alternate alleles to databases containing known functional annotations, which were ANNOVAR^128^ categories, Combined Annotation-Dependent Depletion^129^ scores, RegulomeDB^130^ scores and chromatin state^131,132^:

1. ANNOVAR categories identify SNPs on the basis of their locations with respect to genes, such as exonic, intronic and intergenic, using Ensembl gene definitions.
2. Combined Annotation-Dependent Depletion scores predict deleteriousness, with scores higher than 12.37 suggesting potential pathogenicity^133^.
3. RegulomeDB scores integrate regulatory information from eQTL and chromatin marks, and range from 1a to 7, with lower scores representing more importance for regulatory function.
4. Chromatin states show the accessibility of genomic regions, and were labelled by 15 categorical states on the basis of five chromatin marks for 127 epigenomes in the Roadmap Epigenomics Project^132^, which were H3K4me3, H3K4me1, H3K36me3, H3K27me3 and H3K9me3. For each SNP, FUMA calculated the minimum chromatin state across 127 tissue/cell-type in the Roadmap Epigenomics Project^132^. Categories 1–7 are considered as open chromatin states.

We also used FUMA to annotate independent significant SNPs and their candidate SNPs according to previously reported phenotype associations (*P* < 5 × 10^−5^) in the National Human Genome Research Institute–European Bioinformatics Institute catalogue^134^.

For a significant mvGWAS association in the major histocompatibility complex region (Table 1), we took the most significant individual SNP, rs7781 (*P* = 1.62 × 10^−10^), as the single lead SNP to represent this locus, and annotated it manually.

### SNP-to-gene mapping

SNP-to-gene mapping at significant mvGWAS loci was performed using the default FUMA processes for these three strategies:

1. Positional mapping was used to map SNPs to protein-coding genes on the basis of physical distance (within 10 kb) in the human reference assembly (GRCh37/hg19).
2. eQTL mapping was used to annotate SNPs to genes (that is, when SNP genotypes are associated with variation in gene mRNA expression levels). eQTL mapping was carried out in relation to genes up to 1 Mb away on the basis of four brain-expression data repositories: PsychENCORE^52^, CommonMind Consortium^39^, BRAINEAC^51^ and GTEx v8 Brain^135^. FUMA applied a FDR of 0.05 within each analysis to identify significant eQTL associations.
3. Chromatin interaction mapping was performed to map SNPs to genes on the basis of seven brain-related Hi-C chromatin conformation capture datasets: PsychENCORE EP link (one way)^52^, PsychENCORE promoter anchored loops^39^, HiC adult cortex^46^, HiC foetal cortex^46^, HiC (GSE87112) dorsolateral prefrontal cortex^60^, HiC (GSE87112) hippocampus^60^ and HiC (GSE87112) neural progenitor cell^60^. We further selected only those genes for which one or both regions involved in the chromatin interaction overlapped with a predicted enhancer or promoter region (250 bp up- and 500 bp downstream of the transcription start site) in any of the brain-related repositories from the Roadmap Epigenomics Project^132^, that is, E053 (neurospheres) cortex, E054 (neurospheres) ganglion eminence, E067 (brain) angular gyrus, E068 (brain) anterior caudate, E069 (brain) cingulate gyrus, E070 (brain) germinal matrix, E071 (brain) hippocampus middle, E072 (brain) inferior temporal lobe, E073 (brain) dorsolateral prefrontal cortex, E074 (brain) substantia nigra, E081 (brain) foetal brain male, E082 (brain) foetal brain female, E003 embryonic stem (ES) H1 cells, E008 ES H9 cells, E007 (ES-derived) H1 derived neuronal progenitor cultured cells, E009 (ES-derived) H9 derived neuronal progenitor cultured cells and E010 (ES-derived) H9 derived neuron cultured cells. A FDR of 1 × 10^−6^ was applied to identify significant interactions (default parameter), separately for each analysis.

### Gene-based association analysis

Genome-wide gene-based association analysis was performed using mvGWAS summary statistics as input into MAGMA (v1.08)^65^, using default parameters implemented in FUMA (SNP-wide mean model). This process examines the joint association signals of all SNPs within a given gene (including 50 kb upstream to 50 kb downstream of the gene), while considering the LD between the SNPs. SNPs were mapped to 20,146 protein-coding genes on the basis of National Center for Biotechnology Information build 37.3 gene definitions, and each gene was represented by at least one SNP. We applied a Bonferroni correction for the number of tested genes (*P* < 0.05/20,146).

### Gene-set enrichment analysis

We used MAGMA^65^, again with default settings as implemented in FUMA, to test for enrichment of association within predefined gene sets. This process tests whether gene-based *P* values among all 20,146 genes are lower for those genes within pre-defined functional sets than the rest of the genes in the genome, while correcting for other gene properties such as the number of SNPs. A total of 7,343 gene sets, defined according to Gene Ontology biological processes, were tested from MSigDB version 7.0^69^. In the main text we report the gene sets with *P* values that met Bonferroni correction for multiple testing (*P* < 0.05/7,343).

In addition, we used the list of single closest genes to the 27 lead SNPs arising from mvGWAS (Table 1) as input for gene set enrichment analysis, using the same 7,343 Gene Ontology biological process gene sets, but now on the basis of the hypergeometric test as implemented in GENE2FUNC of FUMA^38^, which is appropriate for gene lists.

Finally, we used the CELL TYPE function (as implemented within FUMA) to test whether lower gene-based association *P* values for brain asymmetry were associated with differential expression levels across cell types, using Bonferroni correction within each separate analysis with respect to each cell-type expression dataset included in FUMA.

### Protein–protein interaction network

We used the Search Tool for the Retrieval of Interacting Genes/Proteins (STRING; http://string-db.org)^68^ for protein network analysis, using as input the names of 57 genes identified through gene-based association analysis, as described above. The STRING dataset includes protein–protein interaction information from numerous sources, including experimental data, publications and computational prediction methods. Only links with medium confidence or higher (confidence score >0.4; default parameter) were retained.

### Developmental stage analysis

Using the gene-based association *P* values for all 20,146 genes genome wide, we used MAGMA (default settings as implemented in FUMA) to examine whether lower gene-based *P* values tended to be found for genes showing relatively higher expression in BrainSpan^71^ gene expression data from any particular ages compared with all other ages, separately for 29 different age groups ranging from 8 postconceptional weeks to 40 years old, and 11 defined developmental stages from early prenatal to middle adulthood. We corrected for multiple testing through a FDR of 0.05 (separately for the two analyses).

### Genetic overlap of brain asymmetry with brain disorders, behavioural and cognitive traits

We applied the iSECA^73^ toolbox that can test for genetic overlap on the basis of per-SNP association *P* values only (mvGWAS does not produce univariate beta coefficient effect size estimates that can be used in standard genetic correlation analysis). We tested for genetic overlap in relation to traits previously reported to associate phenotypically with different aspects of brain structural asymmetry (Main), using GWAS *P* values from previously published, large-scale studies: educational attainment (*n* = 1,131,881)^66^, handedness (*n* = 331,037)^45^, intelligence (*n* = 269,867)^67^, autism spectrum disorder (*n* = 46,350)^78^, attention deficit hyperactivity disorder (*n* = 55,374)^74^ and schizophrenia (*n* = 82,315)^89^. We also tested for genetic overlap in relation to brain intracranial volume (*n* = 32,438)^94^. After LD-based filtering and clumping using default parameters, iSECA tests for pleiotropy between two sets of GWAS results using an exact binomial statistical test at each of 12 *P* value levels: *P* ≤ (0.01, 0.05, 0.1, 0.2, 0.3, 0.4, 0.5, 0.6, 0.7, 0.8, 0.9, 1). The analysis compares the expected and observed overlap in the subsets of SNPs at these levels from two GWAS (144 combinations in total). In other words, iSECA iterates through each of the 12 *P* value levels and counts the number of overlapping variants between two GWAS at each *P* value threshold, and compares that number with the number expected under the null hypothesis of no genetic overlap, using the exact binomial test. iSECA then counts up the number of comparisons with evidence of overlap at a nominally significant level of *P* ≤ 0.05. To assess the significance level of overlap, we generated 1000 datasets through permutations (default parameter), which contained all the possible combinations for a pair of traits, and determined whether the number of levels with nominally significant genetic overlap was significantly more than expected by chance. Finally, Bonferroni correction <0.05 was applied for multiple testing of seven traits. Additionally, iSECA generated Q–Q plots for asymmetry mvGWAS *P* values conditioned on the other trait *P* values (for example, *P* ≤ 0.1, 0.2, 0.3, 0.4, 0.5, 0.75, 1.0) to visualize whether there is an excess of pleiotropic SNPs, which should be visible as a leftward shift of the curve as the *P* value threshold becomes tighter (Supplementary Fig. 8).

### Reporting summary

Further information on research design is available in the Nature Research Reporting Summary linked to this article.

## Supplementary information

Supplementary Information List of Supplementary Tables and Supplementary Figures 1–9.

Reporting Summary

Peer Review Information

Supplementary Tables Supplementary Tables 1–20 in one Excel file with multiple tabs.

## Data Availability

The primary data used in this study are available via the UK Biobank, https://www.ukbiobank.ac.uk. Other publicly available data sources and applications are cited in the Methods section. The GWAS summary statistics are made available online within the GWAS catalogue https://www.ebi.ac.uk/gwas/.

## Code availability

This study used openly available software and codes, specifically GCTA (https://cnsgenomics.com/software/gcta/#GREML), BGENIE (https://jmarchini.org/bgenie/), MetaPhat (https://sourceforge.net/projects/meta-pheno-association-tracer/), FUMA (https://fuma.ctglab.nl/), MAGMA (https://ctg.cncr.nl/software/magma, also implemented in FUMA) and iSECA (https://web.archive.org/web/20190326171900/https://neurogenetics.qimrberghofer.edu.au/iSECA/).

## References

[1] Duboc V, Dufourcq P, Blader P, Roussigne M (2015). Asymmetry of the brain: development and implications. Annu. Rev. Genet..

[2] Kong, X.-Z. et al. Handedness and other variables associated with human brain asymmetrical skew. Preprint at *bioRxiv*10.1101/756395 (2019).

[3] Galaburda AM, Corsiglia J, Rosen GD, Sherman GF (1987). Planum temporale asymmetry, reappraisal since Geschwind and Levitsky. Neuropsychologia.

[4] Herve PY, Crivello F, Perchey G, Mazoyer B, Tzourio-Mazoyer N (2006). Handedness and cerebral anatomical asymmetries in young adult males. Neuroimage.

[5] Kong XZ (2018). Mapping cortical brain asymmetry in 17,141 healthy individuals worldwide via the ENIGMA Consortium. Proc. Natl Acad. Sci. U. S. A..

[6] Guadalupe T (2017). Human subcortical brain asymmetries in 15,847 people worldwide reveal effects of age and sex. Brain Imaging Behav..

[7] Toga AW, Thompson PM (2003). Mapping brain asymmetry. Nat. Rev. Neurosci..

[8] Mazoyer B (2014). Gaussian mixture modeling of hemispheric lateralization for language in a large sample of healthy individuals balanced for handedness. PLoS ONE.

[9] Corballis MC (2017). The evolution of lateralized brain circuits. Front Psychol..

[10] Güntürkün O, Ströckens F, Ocklenburg S (2020). Brain lateralization: a comparative perspective. Physiol. Rev..

[11] Ocklenburg S, Hirnstein M, Beste C, Güntürkün O (2014). Lateralization and cognitive systems. Front. Psychol..

[12] Rentería ME (2012). Cerebral asymmetry: a quantitative, multifactorial, and plastic brain Phenotype. Twin Res. Hum. Genet..

[13] Vingerhoets G (2019). Phenotypes in hemispheric functional segregation? Perspectives and challenges. Phys. Life Rev..

[14] Karolis VR, Corbetta M, Thiebaut de Schotten M (2019). The architecture of functional lateralisation and its relationship to callosal connectivity in the human brain. Nat. Commun..

[15] Postema MC (2019). Altered structural brain asymmetry in autism spectrum disorder in a study of 54 datasets. Nat. Commun..

[16] Postema, M. C. et al. Analysis of structural brain asymmetries in attention-deficit/hyperactivity disorder in 39 datasets. Preprint at *bioRxiv*10.1101/2020.03.03.974758 (2020).

[17] Okada N (2016). Abnormal asymmetries in subcortical brain volume in schizophrenia. Mol. Psychiatry.

[18] Kasprian G (2011). The prenatal origin of hemispheric asymmetry: an in utero neuroimaging study. Cereb. Cortex.

[19] Abu-Rustum RS, Ziade MF, Abu-Rustum SE (2013). Reference values for the right and left fetal choroid plexus at 11 to 13 weeks: an early sign of “developmental” laterality?. J. Ultrasound Med.

[20] McCartney G, Hepper P (1999). Development of lateralized behaviour in the human fetus from 12 to 27 weeks’ gestation. Dev. Med Child Neurol..

[21] Francks C (2019). In search of the biological roots of typical and atypical human brain asymmetry: Comment on “Phenotypes in hemispheric functional segregation? Perspectives and challenges” by Guy Vingerhoets. Phys. Life Rev..

[22] Francks C (2015). Exploring human brain lateralization with molecular genetics and genomics. Ann. N. Y. Acad. Sci..

[23] Vandenberg LN, Lemire JM, Levin M (2013). It’s never too early to get it right: a conserved role for the cytoskeleton in left–right asymmetry. Commun. Integr. Biol..

[24] Vandenberg LN, Levin M (2009). Perspectives and open problems in the early phases of left–right patterning. Semin. Cell Dev. Biol..

[25] Norris DP (2012). Cilia, calcium and the basis of left–right asymmetry. BMC Biol..

[26] Fliegauf M, Benzing T, Omran H (2007). When cilia go bad: cilia defects and ciliopathies. Nat. Rev. Mol. Cell Biol..

[27] Postema MC, Carrion-Castillo A, Fisher SE, Vingerhoets G, Francks C (2020). The genetics of situs inversus without primary ciliary dyskinesia. Sci. Rep..

[28] McManus IC, Martin N, Stubbings GF, Chung EM, Mitchison HM (2004). Handedness and situs inversus in primary ciliary dyskinesia. Proc. Biol. Sci..

[29] Vingerhoets G (2018). Brain structural and functional asymmetry in human situs inversus totalis. Brain Struct. Funct..

[30] Tanaka S, Kanzaki R, Yoshibayashi M, Kamiya T, Sugishita M (1999). Dichotic listening in patients with situs inversus: brain asymmetry and situs asymmetry. Neuropsychologia.

[31] de Kovel CGF (2017). Left–right asymmetry of maturation rates in human embryonic neural development. Biol. Psychiatry.

[32] Ocklenburg S (2017). Epigenetic regulation of lateralized fetal spinal gene expression underlies hemispheric asymmetries. eLife.

[33] Sun T (2005). Early asymmetry of gene transcription in embryonic human left and right cerebral cortex. Science.

[34] Le Guen Y (2020). Enhancer locus in ch14q23.1 modulates brain asymmetric temporal regions involved in language processing. Cereb. Cortex.

[35] Carrion-Castillo A (2020). Genetic effects on planum temporale asymmetry and their limited relevance to neurodevelopmental disorders, intelligence or educational attainment. Cortex.

[36] Yang J (2010). Common SNPs explain a large proportion of the heritability for human height. Nat. Genet..

[37] Lin J, Tabassum R, Ripatti S, Pirinen M (2020). MetaPhat: detecting and decomposing multivariate associations from univariate genome-wide association statistics. Front. Genet..

[38] Watanabe K, Taskesen E, van Bochoven A, Posthuma D (2017). Functional mapping and annotation of genetic associations with FUMA. Nat. Commun..

[39] Fromer M (2016). Gene expression elucidates functional impact of polygenic risk for schizophrenia. Nat. Neurosci..

[40] Berezniuk I (2013). Cytosolic carboxypeptidase 5 removes α- and γ-linked glutamates from tubulin. J. Biol. Chem..

[41] Janke C, Magiera MM (2020). The tubulin code and its role in controlling microtubule properties and functions. Nat. Rev. Mol. Cell Biol..

[42] Bernhardt R, Matus A (1984). Light and electron microscopic studies of the distribution of microtubule-associated protein 2 in rat brain: a difference between dendritic and axonal cytoskeletons. J. Comp. Neurol..

[43] Wiberg A (2019). Handedness, language areas and neuropsychiatric diseases: insights from brain imaging and genetics. Brain.

[44] Partida GC (2020). Genome-wide association study identifies 48 common genetic variants associated with handedness. Nat. Hum. Behav..

[45] de Kovel CGF, Francks C (2019). The molecular genetics of hand preference revisited. Sci. Rep..

[46] Giusti-Rodriguez, P. M. & Sullivan, P. F. Using three-dimensional regulatory chromatin interactions from adult and fetal cortex to interpret genetic results for psychiatric disorders and cognitive traits. Preprint at *bioRxiv*10.1101/406330 (2019).

[47] Lu J (2007). Structure of the FH2 domain of Daam1: implications for formin regulation of actin assembly. J. Mol. Biol..

[48] Ang S-F, Zhao Z-S, Lim L, Manser E (2010). DAAM1 is a formin required for centrosome re-orientation during cell migration. PLoS ONE.

[49] Lancaster OM, Baum B (2014). Shaping up to divide: coordinating actin and microtubule cytoskeletal remodelling during mitosis. Semin. Cell Dev. Biol..

[50] Tischfield MA (2010). Human *TUBB3* mutations perturb microtubule dynamics, kinesin interactions, and axon guidance. Cell.

[51] Ramasamy A (2014). Genetic variability in the regulation of gene expression in ten regions of the human brain. Nat. Neurosci..

[52] Wang D (2018). Comprehensive functional genomic resource and integrative model for the human brain. Science.

[53] Horng S (2009). Differential gene expression in the developing lateral geniculate nucleus and medial geniculate nucleus reveals novel roles for Zic4 and Foxp2 in visual and auditory pathway development. J. Neurosci..

[54] Del Pino I (2020). COUP-TFI/Nr2f1 orchestrates intrinsic neuronal activity during development of the somatosensory cortex. Cereb. Cortex.

[55] Shao W, Halachmi S, Brown M (2002). ERAP140, a conserved tissue-specific nuclear receptor coactivator. Mol. Cell. Biol..

[56] Beales PL, Elcioglu N, Woolf AS, Parker D, Flinter FA (1999). New criteria for improved diagnosis of Bardet–Biedl syndrome: results of a population survey. J. Med Genet.

[57] Zaghloul NA, Katsanis N (2009). Mechanistic insights into Bardet–Biedl syndrome, a model ciliopathy. J. Clin. Investig..

[58] Nenasheva VV, Tarantul VZ (2020). Many faces of TRIM proteins on the road from pluripotency to neurogenesis. Stem Cells Dev..

[59] Himmelfarb M (2004). ITIH5, a novel member of the inter-alpha-trypsin inhibitor heavy chain family is downregulated in breast cancer. Cancer Lett..

[60] Schmitt AD (2016). A compendium of chromatin contact maps reveals spatially active regions in the human genome. Cell Rep..

[61] O’Sullivan ML (2012). FLRT proteins are endogenous latrophilin ligands and regulate excitatory synapse development. Neuron.

[62] Sertie AL (2000). Collagen XVIII, containing an endogenous inhibitor of angiogenesis and tumor growth, plays a critical role in the maintenance of retinal structure and in neural tube closure (Knobloch syndrome). Hum. Mol. Genet.

[63] Feng J, Han Q, Zhou L (2012). Planar cell polarity genes, Celsr1-3, in neural development. Neurosci. Bull..

[64] Al-Mubarak B (2017). Whole exome sequencing reveals inherited and de novo variants in autism spectrum disorder: a trio study from Saudi families. Sci. Rep..

[65] de Leeuw CA, Mooij JM, Heskes T, Posthuma D (2015). MAGMA: generalized gene-set analysis of GWAS data. PLoS Comput. Biol..

[66] Lee JJ (2018). Gene discovery and polygenic prediction from a genome-wide association study of educational attainment in 1.1 million individuals. Nat. Genet..

[67] Savage JE (2018). Genome-wide association meta-analysis in 269,867 individuals identifies new genetic and functional links to intelligence. Nat. Genet..

[68] Szklarczyk D (2017). The STRING database in 2017: quality-controlled protein–protein association networks, made broadly accessible. Nucleic Acids Res..

[69] Liberzon A (2011). Molecular signatures database (MSigDB) 3.0. Bioinformatics.

[70] Grasby KL (2020). The genetic architecture of the human cerebral cortex. Science.

[71] Miller JA (2014). Transcriptional landscape of the prenatal human brain. Nature.

[72] de Kovel CGF, Lisgo SN, Fisher SE, Francks C (2018). Subtle left–right asymmetry of gene expression profiles in embryonic and foetal human brains. Sci. Rep..

[73] Nyholt DR (2014). SECA: SNP effect concordance analysis using genome-wide association summary results. Bioinformatics.

[74] Demontis D (2019). Discovery of the first genome-wide significant risk loci for attention deficit/hyperactivity disorder. Nat. Genet..

[75] Dang LC (2016). Caudate asymmetry is related to attentional impulsivity and an objective measure of ADHD-like attentional problems in healthy adults. Brain Struct. Funct..

[76] Wu ZM (2020). Altered brain white matter microstructural asymmetry in children with ADHD. Psychiatry Res.

[77] Zou H, Yang J (2019). Temporal variability-based functional brain lateralization study in ADHD. J. Atten. Disord..

[78] Grove J (2019). Identification of common genetic risk variants for autism spectrum disorder. Nat. Genet..

[79] Carper RA, Treiber JM, DeJesus SY, Muller RA (2016). Reduced hemispheric asymmetry of white matter microstructure in autism spectrum disorder. J. Am. Acad. Child Adolesc. Psychiatry.

[80] De Fosse L (2004). Language-association cortex asymmetry in autism and specific language impairment. Ann. Neurol..

[81] Floris, D. L. et al. Atypical brain asymmetry in autism–a candidate for clinically meaningful stratification. *Biol. Psychiatry Cogn. Neurosci. Neuroimaging*10.1016/j.bpsc.2020.08.008 (2020). 10.1016/j.bpsc.2020.08.008 33097470

[82] Herbert MR (2005). Brain asymmetries in autism and developmental language disorder: a nested whole-brain analysis. Brain.

[83] Noroozian M, Lotfi J, Gassemzadeh H, Emami H, Mehrabi Y (2002). Academic achievement and learning abilities in left-handers: guilt or gift?. Cortex.

[84] Cheyne CP, Roberts N, Crow TJ, Leask SJ, Garcia-Finana M (2010). The effect of handedness on academic ability: a multivariate linear mixed model approach. Laterality.

[85] Mellet E (2014). Weak language lateralization affects both verbal and spatial skills: an fMRI study in 297 subjects. Neuropsychologia.

[86] Papadatou-Pastou M, Tomprou DM (2015). Intelligence and handedness: meta-analyses of studies on intellectually disabled, typically developing, and gifted individuals. Neurosci. Biobehav Rev..

[87] Prichard E, Propper RE, Christman SD (2013). Degree of handedness, but not direction, is a systematic predictor of cognitive performance. Front Psychol..

[88] Reio TG, Czarnolewski M, Eliot J (2004). Handedness and spatial ability: differential patterns of relationships. Laterality.

[89] Schizophrenia Working Group of the Psychiatric Genomics Consortium. (2014). Biological insights from 108 schizophrenia-associated genetic loci. Nature.

[90] DeLisi LE (1997). Anomalous cerebral asymmetry and language processing in schizophrenia. Schizophr. Bull..

[91] Shenton ME, Dickey CC, Frumin M, McCarley RW (2001). A review of MRI findings in schizophrenia. Schizophr. Res.

[92] Kawasaki Y (2008). Anomalous cerebral asymmetry in patients with schizophrenia demonstrated by voxel-based morphometry. Biol. Psychiatry.

[93] Sun Y, Chen Y, Collinson SL, Bezerianos A, Sim K (2017). Reduced hemispheric asymmetry of brain anatomical networks is linked to schizophrenia: a connectome study. Cereb. Cortex.

[94] Adams HH (2016). Novel genetic loci underlying human intracranial volume identified through genome-wide association. Nat. Neurosci..

[95] Bycroft C (2018). The UK biobank resource with deep phenotyping and genomic data. Nature.

[96] Tee YH (2015). Cellular chirality arising from the self-organization of the actin cytoskeleton. Nat. Cell Biol..

[97] Inaki M, Liu J, Matsuno K (2016). Cell chirality: its origin and roles in left–right asymmetric development. Philos. Trans. R. Soc. Lond. B Biol. Sci..

[98] Okumura T (2008). The development and evolution of left–right asymmetry in invertebrates: lessons from *Drosophila* and snails. Dev. Dyn..

[99] Davison A (2016). Formin is associated with left–right asymmetry in the pond snail and the frog. Curr. Biol..

[100] Steinhauer J, Kalderon D (2006). Microtubule polarity and axis formation in the *Drosophila* oocyte. Dev. Dyn..

[101] McNiven MA, Porter KR (1988). Organization of microtubules in centrosome-free cytoplasm. J. Cell Biol..

[102] Lobikin M (2012). Early, nonciliary role for microtubule proteins in left–right patterning is conserved across kingdoms. Proc. Natl Acad. Sci. U. S. A..

[103] Fan J, Zhang H, Rahman T, Stanton DN, Wan LQ (2019). Cell organelle-based analysis of cell chirality. Commun. Integr. Biol..

[104] Levin M (2006). Is the early left–right axis like a plant, a kidney, or a neuron? The integration of physiological signals in embryonic asymmetry. Birth Defects Res. Part C: Embryo Today.: Rev..

[105] McDowell G, Rajadurai S, Levin M (2016). From cytoskeletal dynamics to organ asymmetry: a nonlinear, regulative pathway underlies left–right patterning. Philos. Trans. R. Soc. Lond. B Biol. Sci..

[106] Bulik-Sullivan BK (2015). LD Score regression distinguishes confounding from polygenicity in genome-wide association studies. Nat. Genet..

[107] Zhou D, Lebel C, Evans A, Beaulieu C (2013). Cortical thickness asymmetry from childhood to older adulthood. Neuroimage.

[108] Roe JM (2021). Asymmetric thinning of the cerebral cortex across the adult lifespan is accelerated in Alzheimer’s disease. Nat. Commun..

[109] Davey Smith G, Ebrahim S (2003). ‘Mendelian randomization’: can genetic epidemiology contribute to understanding environmental determinants of disease?. Int. J. Epidemiol..

[110] de Kovel CGF, Carrion-Castillo A, Francks C (2019). A large-scale population study of early life factors influencing left-handedness. Sci. Rep..

[111] McManus IC (1985). Handedness, language dominance and aphasia: a genetic model. Psychol. Med Monogr. Suppl..

[112] Bishop DVM, Bates TC (2019). Heritability of language laterality assessed by functional transcranial doppler ultrasound: a twin study. Wellcome Open Res.

[113] Janke C, Bulinski JC (2011). Post-translational regulation of the microtubule cytoskeleton: mechanisms and functions. Nat. Rev. Mol. Cell Biol..

[114] Geiger B, Bershadsky A, Pankov R, Yamada KM (2001). Transmembrane crosstalk between the extracellular matrix–cytoskeleton crosstalk. Nat. Rev. Mol. Cell Biol..

[115] Young AI (2019). Solving the missing heritability problem. PLoS Genet..

[116] Mitchell, K. J. *Innate: How the Wiring of Our Brains Shapes Who We Are* (Princeton Univ. Press, 2018).

[117] Aschard H, Vilhjalmsson BJ, Joshi AD, Price AL, Kraft P (2015). Adjusting for heritable covariates can bias effect estimates in genome-wide association studies. Am. J. Hum. Genet..

[118] Sadler TW (2005). Embryology of neural tube development. Am. J. Med. Genet. C. Semin. Med. Genet..

[119] Purcell S (2007). PLINK: a tool set for whole-genome association and population-based linkage analyses. Am. J. Hum. Genet.

[120] Desikan RS (2006). An automated labeling system for subdividing the human cerebral cortex on MRI scans into gyral based regions of interest. Neuroimage.

[121] Alfaro-Almagro F (2018). Image processing and quality control for the first 10,000 brain imaging datasets from UK biobank. Neuroimage.

[122] Kurth F, Gaser C, Luders E (2015). A 12-step user guide for analyzing voxel-wise gray matter asymmetries in statistical parametric mapping (SPM). Nat. Protoc..

[123] Leroy F (2015). New human-specific brain landmark: the depth asymmetry of superior temporal sulcus. Proc. Natl Acad. Sci. U. S. A..

[124] Lee SH, Yang J, Goddard ME, Visscher PM, Wray NR (2012). Estimation of pleiotropy between complex diseases using single-nucleotide polymorphism-derived genomic relationships and restricted maximum likelihood. Bioinformatics.

[125] Hoggart CJ, Clark TG, De Iorio M, Whittaker JC, Balding DJ (2008). Genome-wide significance for dense SNP and resequencing data. Genet. Epidemiol..

[126] Panagiotou OA, Ioannidis JP, Project G-WS (2012). What should the genome-wide significance threshold be? Empirical replication of borderline genetic associations. Int. J. Epidemiol..

[127] The 1000 Genomes Project Consortium. (2015). A global reference for human genetic variation. Nature.

[128] Wang K, Li M, Hakonarson H (2010). ANNOVAR: functional annotation of genetic variants from high-throughput sequencing data. Nucleic Acids Res..

[129] Rentzsch P, Witten D, Cooper GM, Shendure J, Kircher M (2019). CADD: predicting the deleteriousness of variants throughout the human genome. Nucleic Acids Res..

[130] Boyle AP (2012). Annotation of functional variation in personal genomes using RegulomeDB. Genome Res.

[131] Ernst J, Kellis M (2012). ChromHMM: automating chromatin-state discovery and characterization. Nat. Methods.

[132] Roadmap Epigenomics Consortium. (2015). Integrative analysis of 111 reference human epigenomes. Nature.

[133] Kircher M (2014). A general framework for estimating the relative pathogenicity of human genetic variants. Nat. Genet..

[134] MacArthur J (2017). The new NHGRI-EBI catalog of published genome-wide association studies (GWAS catalog). Nucleic Acids Res..

[135] GTEx Consortium. (2015). Human genomics. The Genotype-Tissue Expression (GTEx) pilot analysis: multitissue gene regulation in humans. Science.

