# Supplementary material for: The genetic architecture of structural left–right asymmetry of the human brain
Source: Nat Hum Behav. 2021 Mar 15;5(9):1226–39. doi: 10.1038/s41562-021-01069-w (PMC8455338; doi:10.1038/s41562-021-01069-w)
Supplement: Supplementary file 1 — List of Supplementary Tables and Supplementary Figures 1–9. [file 41562_2021_1069_MOESM1_ESM.pdf]

nature portfolio

https://doi.org/10.1038/s41562-021-01069-w

Supplementary information

The genetic architecture of structural left–right asymmetry of the human brain

In the format provided by the authors and unedited

Supplementary information

Sha et al., The genetic architecture of structural left-right asymmetry of the human brain

List of sections:

1. **List of Supplementary Tables (separate Excel file)**
2. **Supplementary Figures**

Supplementary Tables (separate Excel file)

Supplementary Table 1. The UKB field IDs of brain traits analyzed in our study.

Supplementary Table 2. Population-level regional brain asymmetries (test of mean AI difference from zero, N=32,256).

Supplementary Table 3. Heritabilities of brain regional AIs.

Supplementary Table 4. Correlation coefficients (r values) of phenotypic and genetic correlations between AIs of surface areas.

Supplementary Table 5. P values for phenotypic and genetic correlations between AIs of surface areas.

Supplementary Table 6. Correlation coefficients (r values) of phenotypic and genetic correlations between AIs of cortical thicknesses.

Supplementary Table 7. P values of phenotypic and genetic correlations between AIs of cortical thicknesses.

Supplementary Table 8. Correlation coefficients (r values) of phenotypic and genetic correlations between AIs of subcortical volumes.

Supplementary Table 9. P values of phenotypic and genetic correlations between AIs of subcortical volumes.

Supplementary Table 10. Phenotypic and genetic correlations between AIs of surface area and corresponding cortical thickness for the same regions.

Supplementary Table 11. Effects of the lead SNPs from mvGWAS on each central AI involved in their multivariate association. Post hoc analysis of the corresponding separate left and right measures are also shown.

Supplementary Table 12. Gene mapping at all significant mvGWAS loci based on three strategies (see text).

Supplementary Table 13. Significant genes from gene-based analysis of the mvGWAS results.

Supplementary Table 14. Overlapping annotated genes between brain asymmetry and other traits, with reference to previous GWAS studies (see text).

Supplementary Table 15. Results from MAGMA gene set analysis.

Supplementary Table 16. Results from gene set enrichment analysis based on the closet single genes to mvGWAS lead SNPs (see main Table 1), using GENE2FUNC in FUMA.

Supplementary Table 17. Age-related differential expression of asymmetry-associated genes, in BrainSpan brain samples.

Supplementary Table 18. Genetic overlap between brain asymmetry measures and other traits, which have been reported to show phenotypic associations with brain asymmetry.

Supplementary Table 19. Results in the replication dataset (N=3,600).

Supplementary Table 20. ICD coding for the exclusion of participants with neurological diagnoses.

Supplementary Figures

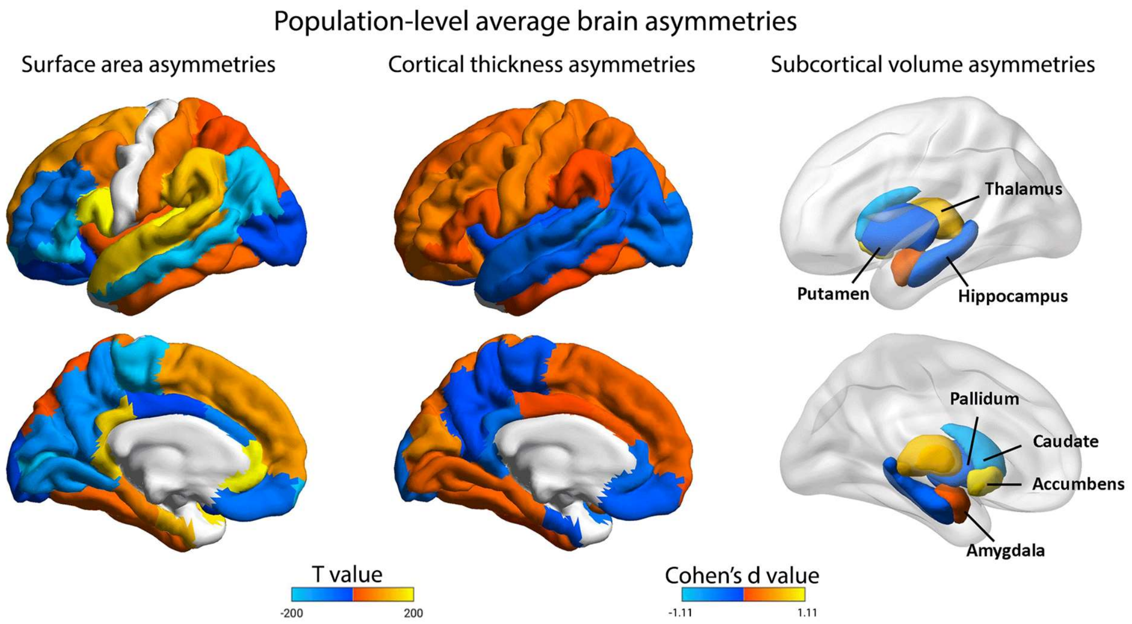

Supplementary Figure 1. Population-level brain regional asymmetries in 32,256 participants.

Orange-yellow indicates significant leftward (left>right) asymmetry, blue indicates significant rightward (left<right) asymmetry. Only regions with significant population-level asymmetries are coloured in the figure.

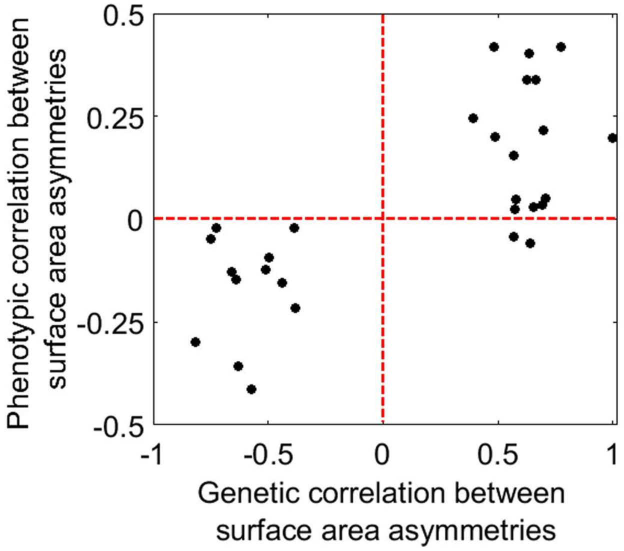

Supplementary Figure 2. Relationship between phenotypic and genetic correlations across pairs of regional surface area AIs (only for pairs of AIs with significant genetic correlations, see Methods).

The plot illustrates that significant genetic correlations were mostly consistent in direction (positive or negative) with the corresponding phenotypic correlations.

Genomic loci affecting brain asymmetries

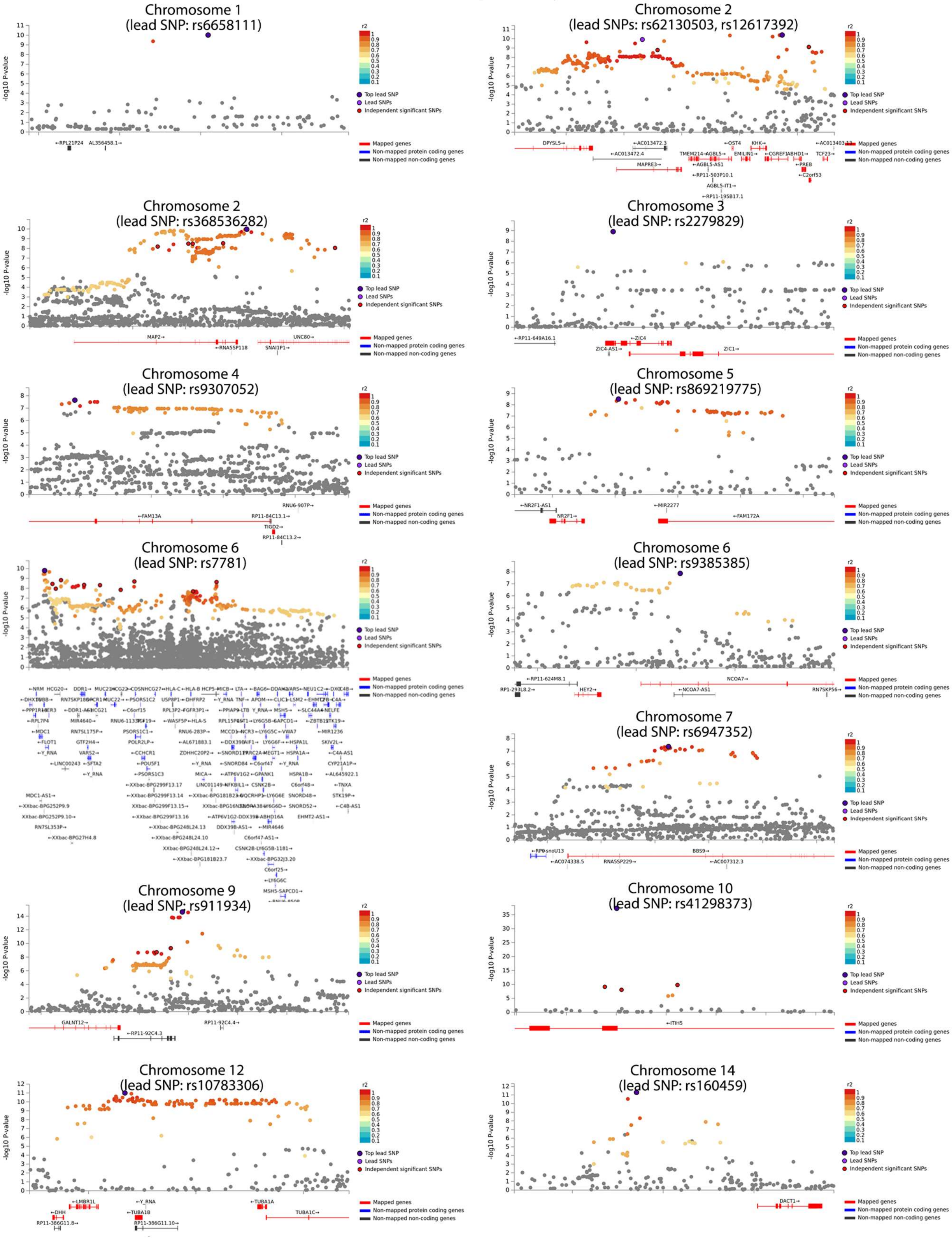

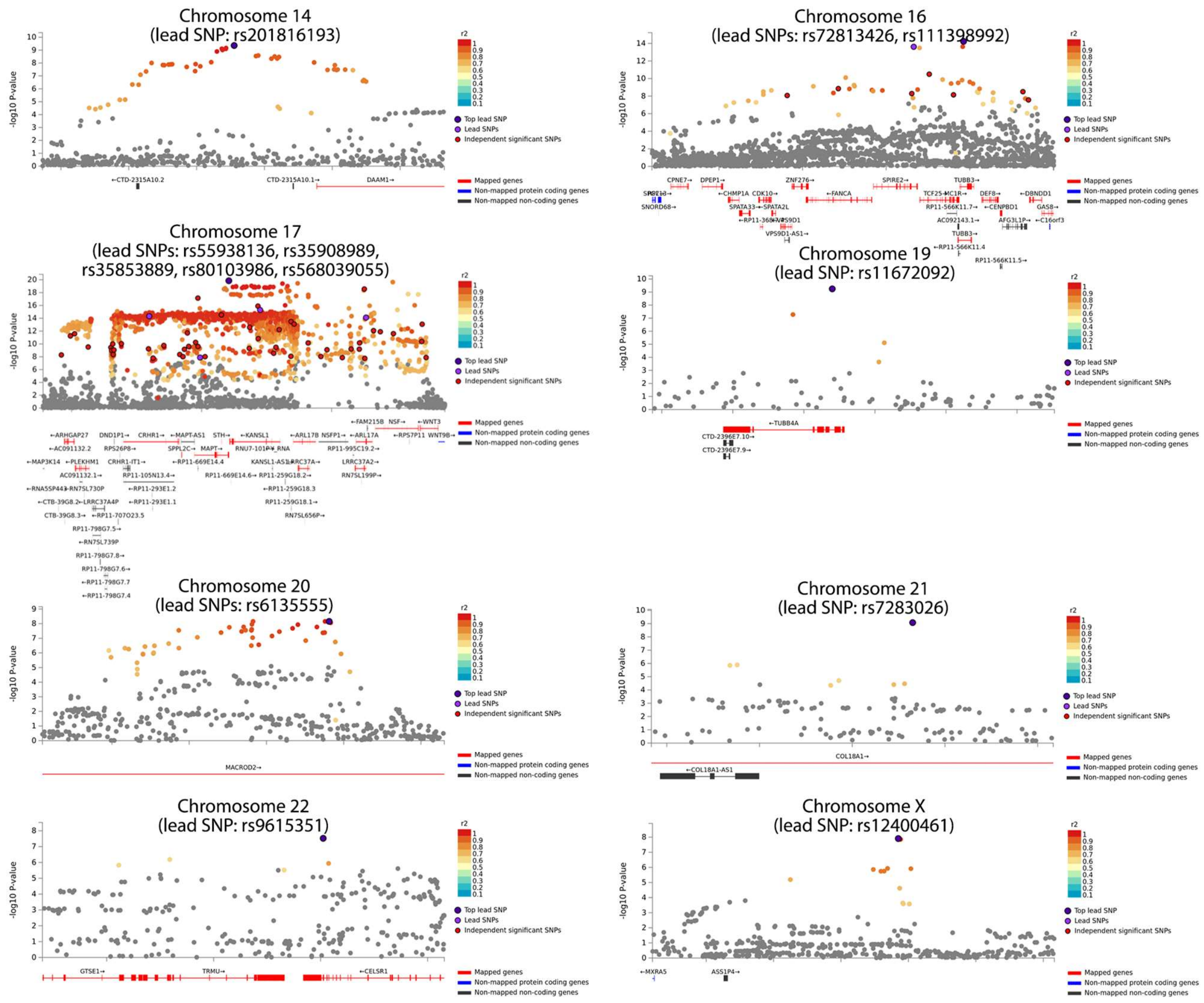

Supplementary Figure 3. Regional association plots for significant loci from the multivariate GWAS for brain asymmetries.

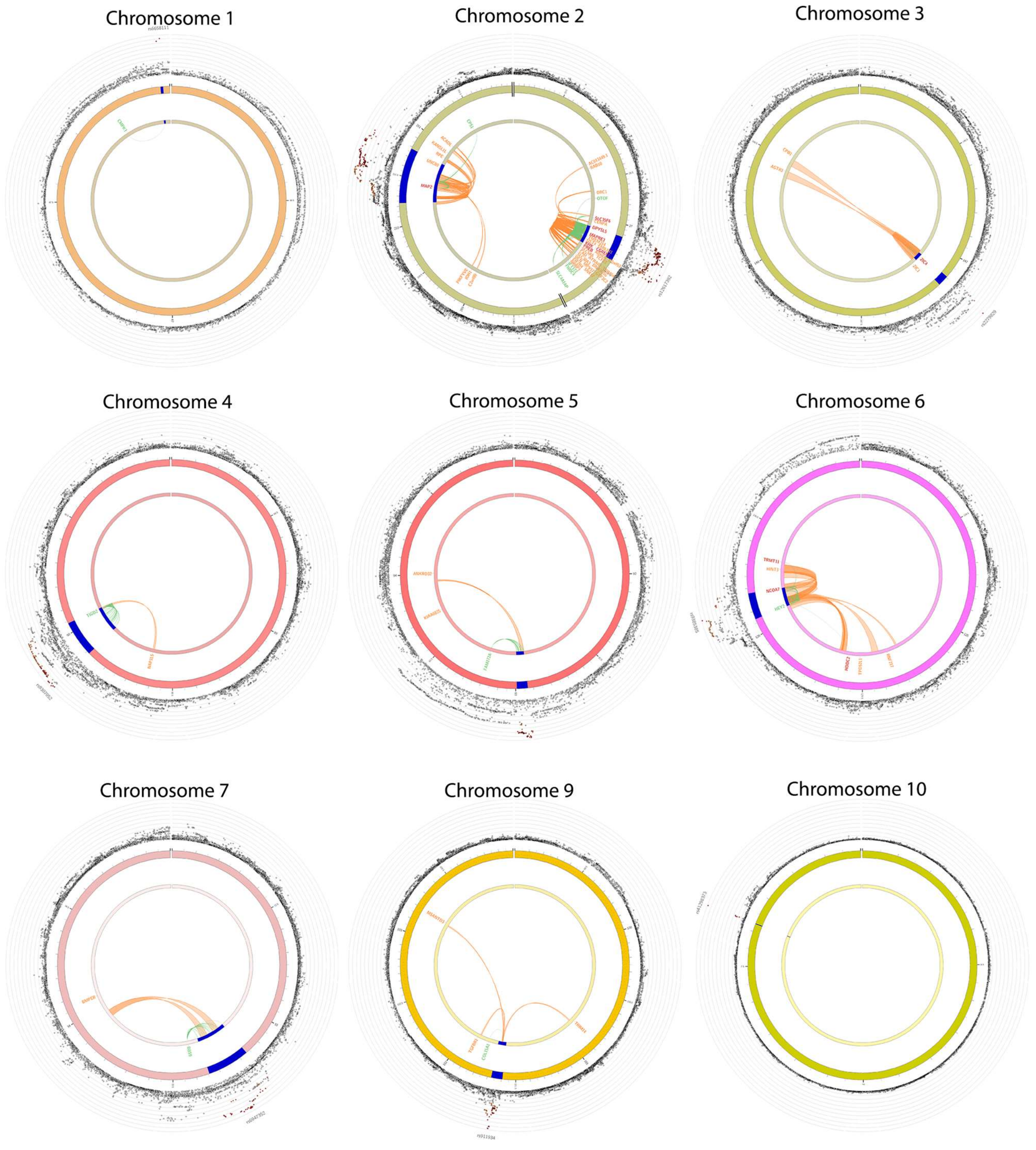
Chromosome 1
Chromosome 2
Chromosome 3
Chromosome 4
Chromosome 5
Chromosome 6
Chromosome 7
Chromosome 9
Chromosome 10

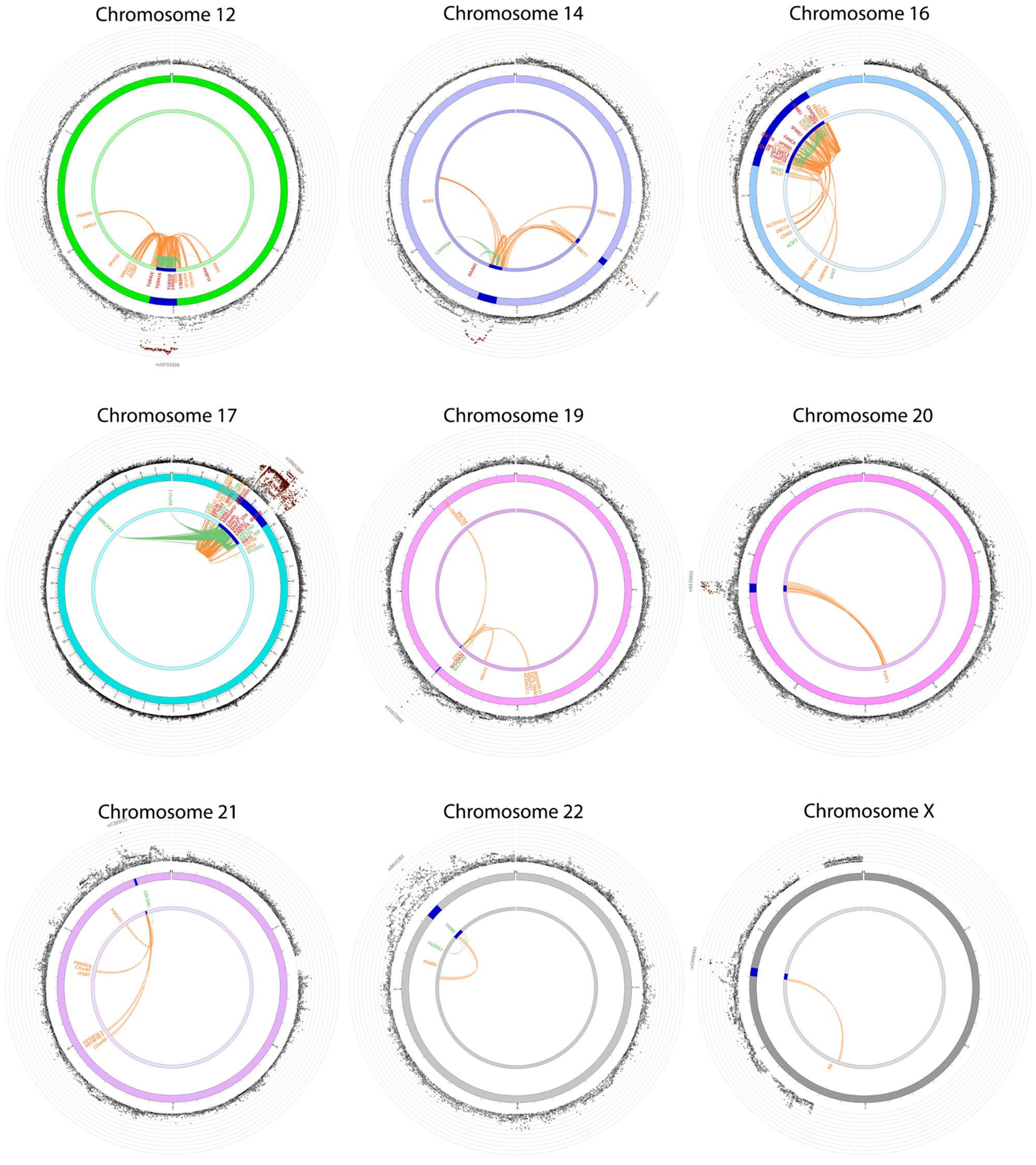

Supplementary Figure 4. Genomic loci, eQTL associations and chromatin interactions identified via multivariate GWAS for brain asymmetries.

Circle plots illustrate the signifciant loci implicated by mvGWAS for brain asymmetries (blue regions), and the genes associated with the loci by eQTL mapping (green), chromatin interactions (orange), or by both strategies (red). The outer layer shows a Manhattan plot containing the log10-transformed p value of each SNP in the mvGWAS of brain asymmetries, with genome-wide significant variants colored by their LD patterns with the lead variant. Empty regions in the Manhattan plot indicate regions where no SNPs with p<0.05 were located.

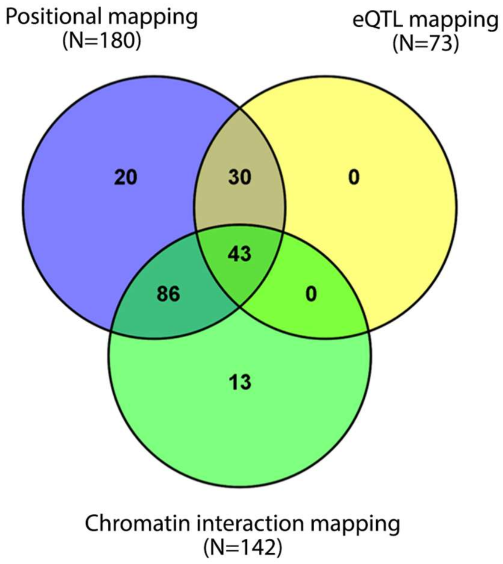

Supplementary Figure 5. Annotated genes at significant mvGWAS loci using three gene mapping strategies in FUMA.

Venn diagram showing the overlap of genes implicated by positional mapping, eQTL mapping and chromatin interaction mapping. One hundred and fifty-nine genes overlapped with at least two strategies.

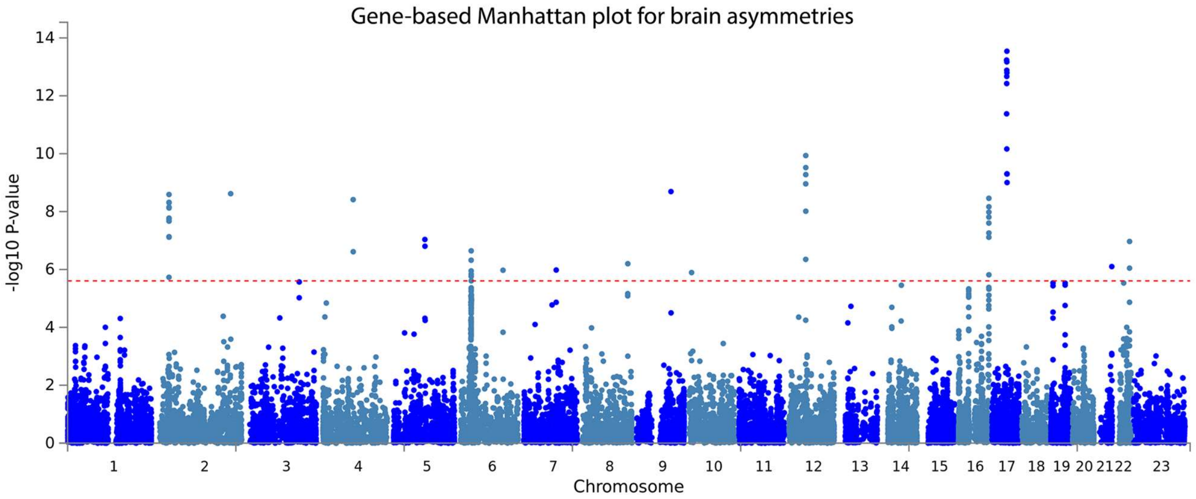

Supplementary Figure 6. Manhattan plot for genome-wide gene-based association analysis of brain asymmetries, using mvGWAS results as input.

The red dashed line indicates the Bonferroni-corrected significance threshold for gene-based analysis (p<0.05/20,146).

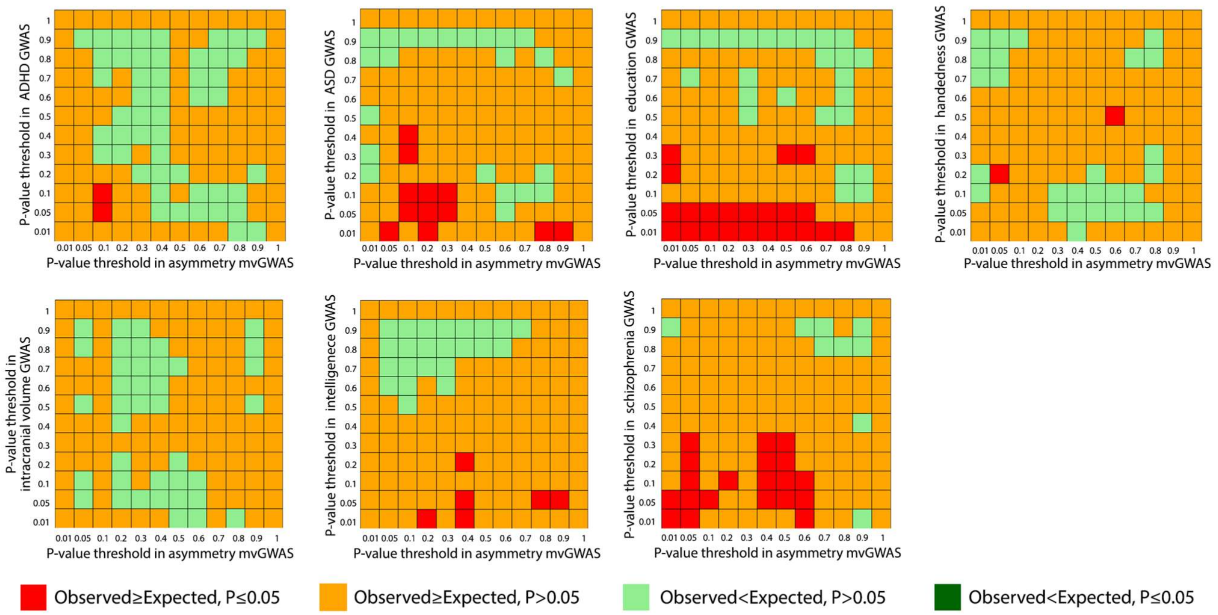

Supplementary Figure 7. Heatmap plots for the tests of pleiotropy with other traits. Genome-wide evidence of pleiotropy between brain asymmetries and other traits, using per-SNP multivariate association P values from the mvGWAS of the current study, in relation to genome-wide association results from previous studies of other traits. Abbreviations: mvGWAS: multivariate genome-wide association scan.

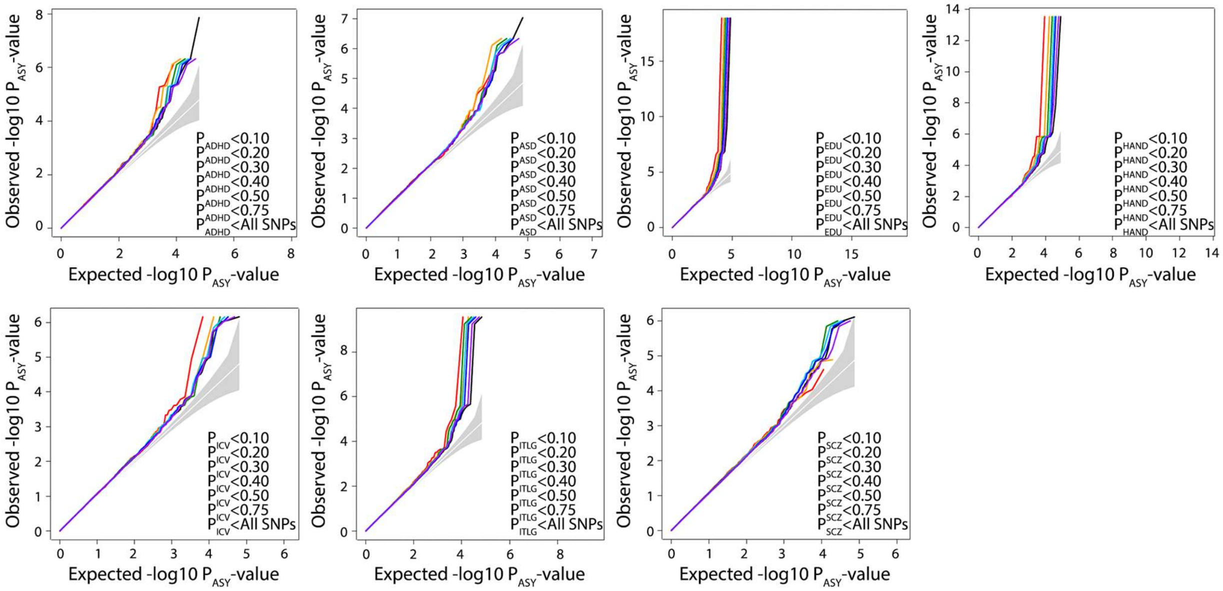

Supplementary Figure 8. Genetic overlaps between brain asymmetry and other traits.
Q-Q plots for mvGWAS per-SNP p-values for brain asymmetries, conditioned on other trait GWAS p-values (at thresholds p⩽0.1, 0.2, 0.3, 0.4, 0.5, 0.75, 1.0) to visualize shared pleiotropic variants. Abbreviations: ADHD: attention deficit hyperactivity disorder; ASD: autism spectrum disorder; ASY: brain asymmetries; EDU: educational attainment; HAND: handedness; ICV: intracranial volume; ITLG: intelligence; SCZ: schizophrenia.

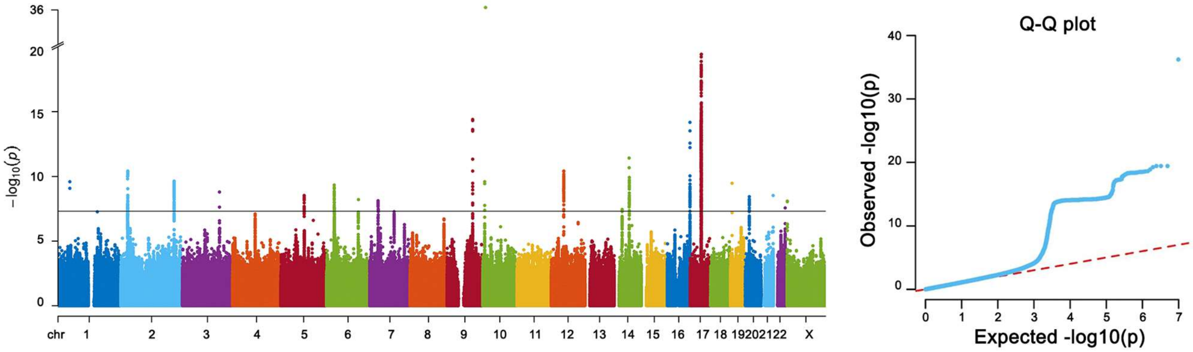

Supplementary Figure 9. Multivariate GWAS analysis of brain asymmetries after excluding 886 participants with neurological diagnoses (leaving 31,370 participants), performed as a sensitivity analysis.

Manhattan plot for multivariate GWAS across asymmetries of surface area, cortical thickness and subcortical volumes. The red dashed line indicates the significance threshold $p<5\times10^{-8}$ (Methods). The Q-Q plot is also shown.
