# Supplementary material for: The genetic architecture of structural left–right asymmetry of the human brain
Source: Nat Hum Behav. 2021 Mar 15;5(9):1226–39. doi: 10.1038/s41562-021-01069-w (PMC8455338; doi:10.1038/s41562-021-01069-w)
Supplement: Supplementary file 2 — Reporting Summary [file 41562_2021_1069_MOESM2_ESM.pdf]

nature research

Corresponding author(s): Clyde Francks

Last updated by author(s): Dec 9, 2020

Reporting Summary

Nature Research wishes to improve the reproducibility of the work that we publish. This form provides structure for consistency and transparency in reporting. For further information on Nature Research policies, see our Editorial Policies and the Editorial Policy Checklist.

Statistics

For all statistical analyses, confirm that the following items are present in the figure legend, table legend, main text, or Methods section.

n/a	Confirmed	
☐	☒	The exact sample size (n) for each experimental group/condition, given as a discrete number and unit of measurement
☐	☒	A statement on whether measurements were taken from distinct samples or whether the same sample was measured repeatedly
☐	☒	The statistical test(s) used AND whether they are one- or two-sided<br>*Only common tests should be described solely by name; describe more complex techniques in the Methods section.*
☐	☒	A description of all covariates tested
☐	☒	A description of any assumptions or corrections, such as tests of normality and adjustment for multiple comparisons
☐	☒	A full description of the statistical parameters including central tendency (e.g. means) or other basic estimates (e.g. regression coefficient) AND variation (e.g. standard deviation) or associated estimates of uncertainty (e.g. confidence intervals)
☐	☒	For null hypothesis testing, the test statistic (e.g. F, t, r) with confidence intervals, effect sizes, degrees of freedom and P value noted<br>*Give P values as exact values whenever suitable.*
☐	☐	For Bayesian analysis, information on the choice of priors and Markov chain Monte Carlo settings
☐	☒	For hierarchical and complex designs, identification of the appropriate level for tests and full reporting of outcomes
☐	☒	Estimates of effect sizes (e.g. Cohen's d, Pearson's r), indicating how they were calculated

Our web collection on statistics for biologists contains articles on many of the points above.

Software and code

Policy information about availability of computer code

Data collection	Data were downloaded from the sources stated in the manuscript.
Data analysis	Publicly available software and versions are stated in the Methods section together with the relevant citations.

For manuscripts utilizing custom algorithms or software that are central to the research but not yet described in published literature, software must be made available to editors and reviewers. We strongly encourage code deposition in a community repository (e.g. GitHub). See the Nature Research guidelines for submitting code & software for further information.

Data

Policy information about availability of data

All manuscripts must include a data availability statement. This statement should provide the following information, where applicable:
- Accession codes, unique identifiers, or web links for publicly available datasets
- A list of figures that have associated raw data
- A description of any restrictions on data availability

The primary data used in this study are available via the UK Biobank website www.ukbiobank.ac.uk . Other publicly available data sources and applications are cited in the Methods section.

Field-specific reporting

Please select the one below that is the best fit for your research. If you are not sure, read the appropriate sections before making your selection.

☒ Life sciences ☐ Behavioural & social sciences ☐ Ecological, evolutionary & environmental sciences

For a reference copy of the document with all sections, see nature.com/documents/nr-reporting-summary-flat.pdf

Life sciences study design

All studies must disclose on these points even when the disclosure is negative.

Sample size	Sample size of >30,000 participants was determined by data availability. This range of sample size has been successfully used in many genome-wide association scan studies of diverse human phenotypes.
Data exclusions	Brain imaging phenotypes were excluded at > 6 SD from the mean to reduce the chance of inlcuding spurious datapoints or extreme points that could bias statistical testing. Single nucleotide polymorphisms were excluded when they had population frequencies below 1%, as statistical testing can be unreliable below this frequency.
Replication	We included a replication sample of >3000 individuals as described in the paper.
Randomization	Not relevant, this was an observational study.
Blinding	Not relevant.

Reporting for specific materials, systems and methods

We require information from authors about some types of materials, experimental systems and methods used in many studies. Here, indicate whether each material, system or method listed is relevant to your study. If you are not sure if a list item applies to your research, read the appropriate section before selecting a response.

Materials & experimental systems

n/a	Involved in the study	
☒	☐	Antibodies
☒	☐	Eukaryotic cell lines
☒	☐	Palaeontology and archaeology
☒	☐	Animals and other organisms
☐	☒	Human research participants
☒	☐	Clinical data
☒	☐	Dual use research of concern

Methods

n/a	Involved in the study	
☒	☐	ChIP-seq
☒	☐	Flow cytometry
☐	☒	MRI-based neuroimaging

Human research participants

Policy information about studies involving human research participants

Population characteristics	The Uk Biobank dataset has been extensively described before. We include demographic information in the paper for the specific subset used in our analysis (i.e. those with post-quality-control brain imaging and genetic data).
Recruitment	The Uk Biobank dataset has been extensively described before.
Ethics oversight	National Research Ethics Service Committee North West-Haydock (reference 11/NW/0382)

Note that full information on the approval of the study protocol must also be provided in the manuscript.

Magnetic resonance imaging

Experimental design

Design type	Structural T1 MRI
Design specifications	1 structural scan per subject
Behavioral performance measures	Not relevant

Acquisition

Imaging type(s)	Structural T1
Field strength	3T
Sequence & imaging parameters	Siemens Skyra 3T and 32-channel RF receive head coil.<br>http://biobank.ndph.ox.ac.uk/showcase/refer.cgi?id=2367
Area of acquisition	Whole brain

Diffusion MRI ☐ Used ☒ Not used

Preprocessing

Preprocessing software	http://biobank.ndph.ox.ac.uk/showcase/refer.cgi?id=2367
Normalization	http://biobank.ndph.ox.ac.uk/showcase/refer.cgi?id=2367
Normalization template	http://biobank.ndph.ox.ac.uk/showcase/refer.cgi?id=2367
Noise and artifact removal	http://biobank.ndph.ox.ac.uk/showcase/refer.cgi?id=2367
Volume censoring	http://biobank.ndph.ox.ac.uk/showcase/refer.cgi?id=2367

Statistical modeling & inference

Model type and settings	Meta-canonical correlation analysis as implemented in MetaPhat: https://www.frontiersin.org/articles/10.3389/fgene.2020.00431/full
Effect(s) tested	Genetic effects on brain regional asymmetry measures, as described in the paper.

Specify type of analysis: ☐ Whole brain ☒ ROI-based ☐ Both

Anatomical location(s): Freesurfer Desikan atlas.

Statistic type for inference (See Eklund et al. 2016)	Region-based measures
Correction	The paper includes various different analyses and the multiple testing approach for each is described explicitly in the methods, either Bonferroni or FDR.

Models & analysis

n/a	Involved in the study
☒	☐ Functional and/or effective connectivity
☒	☐ Graph analysis
☐	☒ Multivariate modeling or predictive analysis

Multivariate modeling and predictive analysis	Meta-canonical correlation analysis was used to test for gene-brain associations as implemented in MetaPhat: https://www.frontiersin.org/articles/10.3389/fgene.2020.00431/full<br>Covariates are defined in the Methods section.
