# Supplementary material for: The genetic architecture of structural left–right asymmetry of the human brain
Source: Nat Hum Behav. 2021 Mar 15;5(9):1226–39. doi: 10.1038/s41562-021-01069-w (PMC8455338; doi:10.1038/s41562-021-01069-w)
Supplement: Supplementary file 3 — Peer Review Information [file 41562_2021_1069_MOESM3_ESM.pdf]

Peer Review Information

Journal: Nature Human Behaviour
Manuscript Title: The genetic architecture of structural left-right asymmetry of the human brain
Corresponding author name(s): Clyde Francks

Editorial Notes:

Reviewer Comments & Decisions:

Decision Letter, initial version:

26th August 2020

Dear Dr Francks,

Thank you once again for your manuscript, entitled "The genetic architecture of structural left-right asymmetry of the human brain", and for your patience during the peer review process.

Your Article has now been evaluated by 2 referees. You will see from their comments copied below that, although they find your work of interest, they have raised various concerns. In light of these comments, we cannot accept the manuscript for publication in its current form, but we would be interested in considering a revised version if you are willing and able to fully address reviewer and editorial concerns.

We hope you will find the referees' comments useful as you decide how to proceed. We are committed to providing a fair and constructive peer-review process. Do not hesitate to contact us if there are specific requests from the reviewers that you believe are technically impossible or unlikely to yield a meaningful outcome.

You will see that the the reviewers request a number of additional analyses, clarification of result reporting, and other analytical revisions. In addition, from an editorial perspective, one of the requirements at Nature Human Behaviour for GWAS studies is the need to provide external validation of findings in at least one replication sample. We would ask you therefore to either conduct an analysis in a replication sample, or to explain why this cannot be done (for example, if you believe there are no suitable replication samples available, please explain why UK Biobank is the only suitable sample.)

Finally, your revised manuscript must comply fully with our editorial policies and formatting requirements. Failure to do so will result in your manuscript being returned to you, which will delay its consideration. To assist you in this process, I have attached a checklist that lists all of our requirements. I have also attached a template manuscript file that exemplifies our policies and formatting requirements. If you have any questions about any of our policies or formatting, please don't hesitate to contact me.

If you wish to submit a suitably revised manuscript we would hope to receive it within 6 months. We understand that the COVID-19 pandemic is causing significant disruptions which may prevent you from carrying out the additional work required for resubmission of your manuscript within this timeframe. If you are unable to submit your revised manuscript within 6 months, please let us know. We will be happy to extend the submission date to enable you to complete your work on the revision.

With your revision, please:

• Include a “Response to the editors and reviewers” document detailing, point-by-point, how you addressed each editor and referee comment. If no action was taken to address a point, you must provide a compelling argument. This response will be used by the editors to evaluate your revision and sent back to the reviewers along with the revised manuscript.

• Highlight all changes made to your manuscript or provide us with a version that tracks changes.

Please use the link below to submit your revised manuscript and related files:

[REDACTED]

Note: This URL links to your confidential home page and associated information about manuscripts you may have submitted, or that you are reviewing for us. If you wish to forward this email to co-authors, please delete the link to your homepage.

Thank you for the opportunity to review your work. Please do not hesitate to contact me if you have any questions or would like to discuss the required revisions further.

Sincerely,
Jamie

Dr Jamie Horder
Senior Editor
Nature Human Behaviour

REVIEWER COMMENTS:

Reviewer #1:
Remarks to the Author:
In this study, the authors investigated genetic factors underlying thickness and volume symmetry of

the human cortex by carrying out a genome-wide association study and subsequent follow-up analyses. They report 21 genomic loci that were significantly associated of which most were new findings. The carry these genomic loci forward into in silico follow-up analyses integrating different types of data. This is an interesting article that sheds new light on a brain phenotype that is associated with a wide variety of outcomes. Their provide consistent evidence of involvement of the cytoskeleton using gene-mapping and gene-set testing.
I have several comments, questions and suggestions to improve the current version of the manuscript:

-Overall, the main text should provide more emphasis why these results are relevant, based on prior evidence of involvement of abnormal lateralization processes in psychiatric disorders (as mentioned in the abstract), instead of summing up results from GWAS follow-up analyses, and how these results be used for further research.

-SNP heritabilities: the main text should mention what software was used to estimate the SNP heritabilities, also for the genetic correlations that follow, instead of only referring to the methods section.

-The estimated SNP heritabilities of the AI indices are in general quite low. It would be of interest to mention the estimated heritability from previous twin study literature for context. Could they also mention the areas with the highest and lowest heritabilities in the main text?

-The authors did not correct for handedness. How much of the genetic signal can be attributed to brain asymmetry differences due to handedness, if this would have been corrected for? (i.e. number of loci, genetic correlation before and after correction)

-Main text: in the result section, the authors provide quite a long list of chromosomal regions (why are these necessary?), lead SNP rs-ids and gene names. I do not see the use of writing out all the data that is also available in table format. Could the authors create some structure in these results (grouping of results) instead of providing a list with results? This would greatly improve the readability of the text.

-The gene-based association testing does not mention the software that was used, this should be stated.

-'Significant enrichment within various microtubule-related sets was also found …' is vague, please provide examples of the top associated sets.

-Figure 2: there is no reason to visualize Manhattan plots as circular plots other than aesthetic reasons. This makes it more difficult to compare heights along each chromosome.

-Figure 4: panel A shows the protein-protein interactions. The authors mention that both experimentally validated interactions are investigated. Is it possible that the authors make clear in the figure which ones are actually experimentally validated interactions and which ones are just based on expert opinion?

-Figure 4: the authors mention that the set of genes show 117 (putative) associations in the STRING

database. The authors mention that this is more than a random set in the proteome. I do not think these findings are that surprising, since any set of genes that is associated with a phenotype is likely to have more interactions that a random set (i.e. gene sets for brain volume, psychiatric disorders, personality, height, intelligence etc.) it would be a fair comparison to compare the set with a set of involved genes of other traits or brain phenotypes.

-Figure 4: the authors state that micro-tubule related genes are located more centrally in the network. Would it be possible to provide the reader with a more quantitative measure for network centrality than using a qualitative term?

-Figure 4: would it be possible to make clear in the figure which protein-protein interactions are actually based on experimentally validated interactions in the STRING database?

-Brain span analyses: the authors again do not mention how these analyses were carried out. Did you use the gene-to-func function embedded in FUMA?

-FUMA has an integrated function of looking into enrichment on the neuronal level. Since the main text mentions associations of genes to neuronal cell-types, it would be interesting whether the authors would actually test this with readily available single-cell data in FUMA.

-The authors used iSECA to estimate genetic overlap with previously associated traits. The merely provide P values for the statistical test, I am missing effect sizes of how much overlap / correlation these was with these traits. Also, how do these relate to more commonly used software to estimate genetic overlap, such as LD Score regression?

-Did the authors estimate overlap with the 2020 Grasby et al. Science paper that carried out GWAS meta-analysis of cortical morphology? The authors mention distinct gene-sets, it would be of interest whether significant overlap with this meta-analysis is present.

-Any single-sample GWAS should provide some measure of external validation of their findings in another sample (i.e. the reproducibility of loci or explained variance by a polygenic score etc.) since the use of only UK Biobank data is prone to overfitting.

-For transparency and to allow other groups to make optimal use of the results, do the authors plan to make the GWAS summary statistics publicly available?

Reviewer #2:
Remarks to the Author:
The authors conducted a genome-wide association study (GWAS) of left-right asymmetry of various brain regions. The subjects were 32,256 participants in the UK Biobank. Despite the relatively small sample size by GWAS standards and the low GCTA heritabilities of the asymmetries, the authors were able to detect 21 genome-wide significant loci associated with different asymmetries. They employed MetaPhat, a new GWAS tool that implements canonical correlation analysis and uses a stepwise procedure to identify the components of the multivariate phenotype contributing to a significant canonical correlation. Using the enrichment tool MAGMA, they found that genes with low association p-

value SNPs were unusually likely to be implicated in microtubule function. Despite what might have seemed a low statistical power to detect such enrichment, this finding seems statistically secure and is thus quite impressive and intriguing.

Some readers may be skeptical of the canonical correlation analysis. This may seem like a way to get something out of nothing. Without studying the specific method more closely, I think it is warranted to be skeptical of whether a significant multivariate hit can be followed up to determine the contributing components with an overwhelming degree of confidence. But in most of the downstream analyses, only the multivariate results are used, and I think the resulting inferences can be trusted. Readers can inspect Supplementary Table 11 to see the univariate associations that seem to be driving each multivariate hit, and this provides some transparency.

I recommend the publication of this paper, upon a few minor revisions and clarifications.

p. 5, lines 103-104: "Seventy-two of the 112 genes have been reported to associate with educational attainment"

The cited paper used two different gene-prioritization tools, DEPICT and MAGMA (the one used by these authors). The DEPICT genes were nearly a subset of the MAGMA genes. Nevertheless, it will helpful to state which of the two lists of prioritized genes in the cited paper were checked for overlap with the 112 asymmetry genes.

pp. 13-14, lines 328-334: There needs to be clarification of what qualifies a gene to "belong" to a particular stage. I.e., what makes a gene an "early prenatal" gene or a "late childhood" gene? Right now the authors say "differential mRNA expression (significantly higher gene expression)," but this is too vague.

Supplementary Figure 10: I suggest removing this figure. I don't think it is referred to in the main text. (The supplementary information refers to a "Supplementary Figure 14," but there are only 10 supplementary figures.) I think this kind of annotation is only informative if the SNPs have a good chance of being causal. Here, annotation of lead SNPs and all SNPs in LD r^2 > 0.6 is too inclusive.

Author Rebuttal to Initial comments

Author responses to reviewer comments

Sha *et al.*, The genetic architecture of structural left-right asymmetry of the human brain

Authors: We thank the editor and reviewers for helpful comments on the manuscript.

<u>Editor</u>
... one of the requirements at Nature Human Behaviour for GWAS studies is the need to provide external validation of findings in at least one replication sample. We would ask you therefore to

either conduct an analysis in a replication sample, or to explain why this cannot be done (for example, if you believe there are no suitable replication samples available, please explain why UK Biobank is the only suitable sample.)

Authors: We have added additional results in the "Validation of lead SNPs associated with brain asymmetry" section (page 8):

"To achieve a reasonable level of genetic homogeneity in our mvGWAS, we had excluded any individuals not annotated as having 'white British ancestry' (through a combination of self-report and clustering based on principal components that capture major axes of genotypic diversity[94] (Methods)). The UK Biobank includes additional participants who self-identify as being 'white', but who did not self-identify as British, or did not cluster genetically with the bulk of the 'white British' ancestry participants (Methods). After applying the same quality control criteria to these additional participants as in our discovery mvGWAS (except with respect to ancestry), and imposing the extra criterion that relatedness kinship coefficients should not be greater than 0.0442 with any participants from the discovery mvGWAS, data were available from 3,600 participants for an independent replication set. We tested each of the 27 lead SNPs from the mvGWAS in the replication set, using the same approach as the mvGWAS analysis, except that 40 genetic principle components were used as covariates to control for the greater degree of ancestral diversity in the replication set.

Ten of the 27 independent lead SNPs from the discovery mvGWAS showed association p values < 0.05 in multivariate testing in the replication set (Supplementary Table 19). The combined p value of the remaining 17 lead SNPs was $p=3.3\times10^{-4}$ in the replication set (calculated by Stouffer's method), which we confirmed by permutation with respect to 10,000 repeat random samplings of 17 SNPs from the whole genome in the replication set (permutation-based $p=4\times10^{-4}$). This indicates that the limited sample size of the replication set, compared to the discovery set, did not provide adequate power to replicate at the level of some individual SNPs, but that in combination there was evidence for replication. Moreover, among the 17 SNPs that showed p>0.05 in multivariate testing in the replication set, some showed association p<0.05 in univariate testing of the specific central traits identified for those SNPs in the discovery mvGWAS (Supplementary Table 19). It is also worth noting that four of these 17 SNPs (or SNPs in high linkage disequilibrium with them) have been reported to associate with left-handedness at a genome-wide significant level[42] (see above for details), which is an additional form of validation with respect to a phenotype related to brain asymmetry. As also mentioned above, the high degree of functional clustering of genes identified through gene-based association testing is another form of support for the association results in the mvGWAS."

Finally, your revised manuscript must comply fully with our editorial policies and formatting requirements.

Authors: We have formatted the revised paper according to the template provided, and completed the checklist.

• Highlight all changes made to your manuscript or provide us with a version that tracks changes.

Authors: We have highlighted the relevant points in the manuscript with reference to this document.

<u>Reviewer #1:</u>
In this study, the authors investigated genetic factors underlying thickness and volume symmetry of the human cortex by carrying out a genome-wide association study and subsequent follow-up analyses. They report 21 genomic loci that were significantly associated of which most were new findings. The carry these genomic loci forward into in silico follow-up analyses integrating different types of data. This is an interesting article that sheds new light on a brain phenotype that is associated with a wide variety of outcomes. Their provide consistent evidence of involvement of the cytoskeleton using gene-mapping and gene-set testing.
I have several comments, questions and suggestions to improve the current version of the manuscript:

Authors: We thank the reviewer for these valuable comments and suggestions.

-Overall, the main text should provide more emphasis why these results are relevant, based on prior evidence of involvement of abnormal lateralization processes in psychiatric disorders (as mentioned in the abstract), instead of summing up results from GWAS follow-up analyses, and how these results be used for further research.

Authors: The main inference from our results on genetic overlap is that genes affecting brain asymmetry are also involved in susceptibility to autism and schizophrenia. We discuss this observation with reference to previous literature and causal versus correlational models, and how these might be distinguished in the future. However, we have not reduced the emphasis in the main text on the other major aspect of this research, i.e. that brain laterality is particularly affected by microtubule and embryonically-expressed genes, which is highly relevant to understanding the genetic and developmental foundations of brain laterality, regardless of any connection to disorders.
The discussion section on the genetic overlap with psychiatric disorders now reads like this (page 11):

“We found significant genetic overlaps of brain asymmetry with autism, schizophrenia and educational attainment, which indicates that genes affecting brain asymmetry also influence these traits. This is in line with literature that has shown phenotypic associations between altered brain asymmetry and these traits (see Introduction), and indicates that such phenotypic associations are contributed to an extent by genetic factors. As we found that brain asymmetry-related genes tend to be especially highly expressed in the embryonic brain, then it seems likely that the genetic overlap of brain asymmetry and disorders reflects a genetic susceptibility to alterations of early neurodevelopment away from the typical trajectory. However, brain asymmetry continues to develop throughout the lifespan[105,106], and the UK Biobank consists of middle to older age adults, so that our mvGWAS may have also identified genetic factors that affect brain asymmetrical changes later in life.
Further research, for example using Mendelian Randomization[107], will be needed to understand whether brain asymmetries mediate gene-disorder associations in a causal sense, or whether altered brain asymmetry and disorder susceptibility are two distinct consequences arising from a partly overlapping genetic basis. It will also be important to map, on a brain-regional basis, which aspects of asymmetry show the strongest genetic overlaps with disorder susceptibility. Larger imaging-genetic datasets may be needed to support causal mediation and mapping studies with respect to disorders, as the present genetic association analysis was based on brain-wide asymmetry (albeit in a multivariate context).”

-SNP heritabilities: the main text should mention what software was used to estimate the SNP heritabilities, also for the genetic correlations that follow, instead of only referring to the methods section.

Authors: Done (page 4)

-The estimated SNP heritabilities of the AI indices are in general quite low. It would be of interest to mention the estimated heritability from previous twin study literature for context. Could they also mention the areas with the highest and lowest heritabilities in the main text?

Authors: We have added the information about highest and lowest heritabilites to the main text (page 4), and made an overall comparison with previous twin studies in the Discussion (page 11), together with some explanation of why twin-based heritabilities are often slightly higher than SNP-based heritabilities (SNP-based heritability is based on only one class of genomic variation, while twin studies can overestimate heritability when certain assumptions are not met).

In the "Heritabilities and genetic correlations of brain regional asymmetry measures" section of Results (page 4):
"Forty-two AIs showed significant SNP-based heritabilities (FDR-corrected p<0.05), i.e. 28 of the surface area AIs, 8 cortical thickness AIs, and 6 subcortical volume AIs (Fig. 1A, Supplementary Table 3), ranging from 2.2% for the AI of entorhinal cortical thickness, to 9.4% for the AI of superior temporal surface area. The overall pattern was consistent with previous twin-based heritability analyses[5,6]."

In the Discussion (page 11):
"Many of the brain asymmetries were strong and directional at the population level, but their heritabilities were generally low, ranging up to 10%. This suggests that developmental mechanisms for brain asymmetry are tightly constrained and largely genetically invariant in the population, and that environmental factors and/or developmental randomness are responsible for most variability[43,108-110]. A cytoskeleton-based origin of brain asymmetry would fit this scenario, as the cytoskeleton is essential for various fundamental functions in cellular biology, beyond axis formation[111,112]. Previous twin- and family-based analyses[5,6] have reported heritabilities up to roughly 25% for some of the same asymmetry measures that we analyzed in the present study, with an overall similar regional pattern, i.e. higher heritabilities particularly for regions important in the language system (e.g. superior temporal cortex) and limbic system (e.g. medial temporal and cingulate cortex). Twin-based heritability is often measured to be higher than SNP-based heritability, which may be expected because SNPs are just one class of genetic variation, and also because twin studies can overestimate heritability when certain assumptions are not fully met[113]. As twin studies have not indicated effects of shared environment on brain aymmetries[5,6], then early developmental randomness is likely to cause most variation[114]."

-The authors did not correct for handedness. How much of the genetic signal can be attributed to brain asymmetry differences due to handedness, if this would have been corrected for? (i.e. number of loci, genetic correlation before and after correction)

Authors: It is generally not advisable in genetic analysis to correct for covariates which are themselves partly heritable (such as handedness), as collider bias can induce biased or spurious genetic effects on the target trait (in this case brain asymmetry).

- **Aschard et al. Adjusting for Heritable Covariates Can Bias Effect Estimates in Genome-Wide Association Studies** doi: 10.1016/j.ajhg.2014.12.021

Handedness cannot therefore be treated safely as a confound variable when analyzing brain asymmetry. We are interested in any genetic effects on brain asymmetry, regardless

of whether they might also be shared with other traits such as handedness. Having identified genetic effects on brain asymmetry, we then report post hoc whether they have been reported as significant in previous GWAS of handedness in over 1 million people (five of our identified variants associated with brain asymmetry were reported to associate with handedness by Partida et al. (biorxiv preprint)). As reported in the paper, apart from this small number of individual loci, we did not find a significant genome-wide genetic overlap between brain asymmetry and handedness.

We have now explained in the revised Discussion (page 12):
"We did not correct for handedness as a covariate in our genetic analyses, as it is generally not advisable to correct for covariates which are themselves partly heritable. This is because biased genetic effects can arise with respect to the target trait[115] (in this case brain asymmetry). Handedness cannot therefore be treated safely as a confound variable when analyzing brain asymmetry. We were interested in any genetic effects on brain asymmetry, regardless of whether they might also be shared with other traits such as handedness. Having identified genetic effects on brain asymmetry, we then queried post hoc whether they have been reported as significant in previous GWAS of handedness in over 1 million people[42]. We did not observe a significant genetic overlap between structural brain asymmetry and handedness at the genome-wide level, which again may be due to the relatively low SNP-based heritabilities of these traits, in combination with limited statistical power in the present sample size for this kind of analysis. However, five individual SNPs affecting brain asymmetry and handedness were identified, which suggests that a significant genome-wide overlap might be detected when using a larger dataset in the future."

-Main text: in the result section, the authors provide quite a long list of chromosomal regions (why are these necessary?), lead SNP rs-ids and gene names. I do not see the use of writing out all the data that is also available in table format. Could the authors create some structure in these results (grouping of results) instead of providing a list with results? This would greatly improve the readability of the text.

Authors: We have re-organized into three distinct paragraphs, the first for summarizing the significant variants and associated brain traits in mvGWAS analysis, the second about loci that implicate microtubule-related genes specifically, and the third about all other loci associated with neural and brain development (page 5). It is important to retain this information in text form locus-by-locus, because it distills a selection of particularly notable biological annotations for each of the 27 independent lead SNPs that are associated with brain asymmetry, whereas the supplementary tables and figures have more detailed information of different types and are not necessarily organized by loci. These paragraphs

also cite many primary references in which relevant information is reported for each locus. Inserting citations may not work in table form.

-The gene-based association testing does not mention the software that was used, this should be stated.

Authors: MAGMA v1.08 was used to perform gene-based association analysis. We have clarified this in the Methods (page 18).

-'Significant enrichment within various microtubule-related sets was also found …' is vague, please provide examples of the top associated sets.

Authors: Significant enrichment within various microtubule-related sets, such as 'microtubule_cytoskeleton_organization' ($p=2.19\times10^{-7}$) and 'microtubule_based_process' ($p=2.36\times10^{-6}$), was also found when using the list of single closest genes (Table 1) to the 27 lead SNPs (Supplementary Table 16). We have named the top sets in the main text (page 7).

-Figure 2: there is no reason to visualize Manhattan plots as circular plots other than aesthetic reasons. This makes it more difficult to compare heights along each chromosome.

Authors: We have made new Manhattan plots in the traditional style in Figure 2 and Supplementary Figure 9.

-Figure 4: panel A shows the protein-protein interactions. The authors mention that both experimentally validated interactions are investigated. Is it possible that the authors make clear in the figure which ones are actually experimentally validated interactions and which ones are just based on expert opinion?

Authors: This figure is generated by the STRING software. Edges between nodes represent different types of protein-protein associations provided in the STRING database. We have added details to the figure legend on the color coding of the different types of interaction (page 36):
"Edges between nodes represent different types of protein-protein interactions according to the STRING database (Methods), including known interactions (turquoise and dark purple represent interactions identified by curated databases and biological experiments, respectively), predicted interactions (green, red and blue represent interactions predicted by gene neighborhood, gene fusions and gene co-occurrence, respectively) and others (yellow, black and light purple represent interactions determined by textmining, co-expression and protein homology, respectively)."

-Figure 4: the authors mention that the set of genes show 117 (putative) associations in the STRING database. The authors mention that this is more than a random set in the proteome. I do not think these findings are that surprising, since any set of genes that is associated with a phenotype is likely to have more interactions that a random set (i.e. gene sets for brain volume, psychiatric disorders, personality, height, intelligence etc.) it would be a fair comparison to compare the set with a set of involved genes of other traits or brain phenotypes.

Authors: This analysis primarily provides a validation of our genetic association data, as the clustering of genes implicated by genetic association analysis into an interacting set, at a level far exceeding chance, is a strong indicator that the genetic association data themselves are not merely random noise. In addition, the protein-protein interaction analysis helps to increase biological understanding of the genetic contributions to brain asymmetry.
We do not know the extent to which it is a general principle that any set of genes associated with a phenotype is likely to have more protein-protein interactions than a random set. This is an empirical question that would require gathering GWAS summary statistics from many traits (certainly an interesting idea and study, but not the goal of ours). The comparison with our data might only be meaningful for traits with similar numbers of associated genes, in which case the particular genetic architecture of those traits would likely determine the outcome of the comparison, rather than any general principal of complex trait genetic architecture.

We have clarified the issue in the revised Results section "Functional annotations of genomic loci associated with brain asymmetry" (page 7):
"For the proteins encoded by the 57 genes, there were 80 known or putative pairwise interactions in the STRING database[66], compared to 8 interactions expected for a random set of this size from the whole proteome ($p<1\times10^{-16}$). This observation supports the validity of our mvGWAS association findings, as random noise would not lead to such functional clustering,

-Figure 4: the authors state that micro-tubule related genes are located more centrally in the network. Would it be possible to provide the reader with a more quantitative measure for network centrality than using a qualitative term?

Authors: We do not have a quantitative analysis of this, so we have changed the relevant description to note only that some specific microtubule-related genes link clusters together, with reference to the relevant figure (page 7):
"Microtubule-related genes (e.g. *MAP2, MAPT, SPIRE2* and *TUBA1A*) linked different clusters together in the largest protein interaction network (Fig. 4A)."

-Figure 4: would it be possible to make clear in the figure which protein-protein interactions are

actually based on experimentally validated interactions in the STRING database?

Authors: This figure is generated by the STRING software. Edges between nodes represent different types of protein-protein associations provided in the STRING database. We have added details to the figure legend on the colour coding of the different types of interaction (page 36).
"Edges between nodes represent different types of protein-protein interactions according to the STRING database (Methods), including known interactions (turquoise and dark purple represent interactions identified by curated databases and biological experiments, respectively), predicted interactions (green, red and blue represent interactions predicted by gene neighborhood, gene fusions and gene co-occurrence, respectively) and others (yellow, black and light purple represent interactions determined by textmining, co-expression and protein homology, respectively)."

-Brain span analyses: the authors again do not mention how these analyses were carried out. Did you use the gene-to-func function embedded in FUMA?

Authors: Yes, we used the embedded function in FUMA. We have made sure to note this in the Methods (page 19).

-FUMA has an integrated function of looking into enrichment on the neuronal level. Since the main text mentions associations of genes to neuronal cell-types, it would be interesting whether the authors would actually test this with readily available single-cell data in FUMA.

Authors: There was no significant genome-level enrichment of association signals with respect to cell-types. In the Results, we describe that some of the individual genes at the 21 associated genomic loci have shown particularly high expression in the context of certain tissues, cell types or subcellular structures, but this does not necessarily imply significant enrichment at the whole-genome level. Thus the genome-wide testing does not impact on the validity of observations made with respect to the top loci from the GWAS. We have added the relevant methods and results in the revised manuscript:

In the "Functional annotations of genomic loci associated with brain asymmetry" section of Results (page 7):
"We observed no statistically significant relation of our gene-based association p values with differential expression across cell types (Methods)."

In the "Gene-set enrichment analysis" of Methods (page 19):
"Finally, we used the CELL TYPE function (as implemented within FUMA) to test whether lower gene-based association p values for brain asymmetry were associated with

differential expression levels across cell types, using Bonferroni correction within each separate analysis with respect to each cell-type expression dataset included in FUMA."

-The authors used iSECA to estimate genetic overlap with previously associated traits. The merely provide P values for the statistical test, I am missing effect sizes of how much overlap / correlation these was with these traits. Also, how do these relate to more commonly used software to estimate genetic overlap, such as LD Score regression?

Authors: We used multivariate GWAS analysis to achieve data reduction and increase statistical power, for exploiting genetic covariance between 42 brain asymmetry measures. In this framework we perform one association test for each genetic variant simultaneously across all 42 asymmetry measures, rather than performing 42 separate tests per genetic variant. This approach has the advantage that we can then perform downstream analyses, such as functional enrichment and genetic overlap with disorders, using the results from one multivariate GWAS rather than separately for 42 GWAS of regional asymmetry measures. The latter approach would be cumbersome and difficult to interpret and/or underpowered with respect to correction for multiple testing. A consequence of our multivariate approach is that it does not yield association effect sizes across different traits, and therefore the results cannot be used for standard genetic correlation analyses, such as performed with LD score regression. This is why we refer to genetic overlap rather than genetic correlation, and is why we chose iSECA to investigate this, based only on P values and not univariate effect sizes. We have explained in more detail in the revised version:

In the "Multivariate genome-wide association analysis (mvGWAS)" section of Results (page 5):
"A multivariate approach had the dual advantages of achieving data reduction and increasing statistical power compared to running 42 separate univariate GWAS."

In the Discussion (page 10):
"In this study we identified genetic loci that are associated with 42 heritable aspects of brain asymmetry through a multivariate, brain-wide approach. A multivariate approach can boost statistical power while achieving data reduction, compared to separate univariate analyses of individual brain traits[37]. A single set of genome-wide association results, pertaining simultaneously to multiple aspects of brain asymmetry, was then taken forward into functional annotation and downstream analyses, such as testing for genetic overlaps with other traits. The multivariate approach therefore helped to detect and interpret key aspects of the genetic architecture of brain asymmetry, without the noise inherent in repeat univariate testing. An important challenge remained to identify the particular brain traits that drove the multivariate associations at each locus, which was achieved in MetaPhat[37] by

decomposing associations into sets of 'central' traits based on the Bayesian Information Criterion and univariate p-value statistics.
A consequence of the multivariate approach is that it does not yield univariate association effect sizes, and therefore mvGWAS results cannot be used for standard genetic correlation analyses, such as is performed with LD score regression[104]. Therefore, we used iSECA[72] to explore the genetic overlap of brain asymmetry with neurodevelopmental disorders, behavioral and psychological traits."

-Did the authors estimate overlap with the 2020 Grasby et al. Science paper that carried out GWAS meta-analysis of cortical morphology? The authors mention distinct gene-sets, it would be of interest whether significant overlap with this meta-analysis is present.

Authors: Grasby et al. worked with bilateral structural measures that were generally more heritable than the asymmetry measures that we have analyzed. They chose to perform mass univariate GWAS analyses in their study. In our study, we performed multivariate GWAS analysis to identify genetic loci that are associated with different aspects of brain asymmetry. We are unsure whether it would be informative to test for genetic overlaps between our multivariate results for brain asymmetry and each of Grasby's univariate sets of results for separate regional measures. We have included a test for genetic overlap with a previous ENIGMA consortium GWAS for overall brain size, which seems appropriate with respect to our multivariate approach that captures brain-wide asymmetry in a single genetic analysis. The results showed no significant genetic overlap of brain size with brain asymmetry, which indicates that the genetic architecture of brain asymmetry is distinct from brain size. Moreover, enrichment in microtubule-related sets was not reported in Grasby's GWAS of bilaterally averaged cortical surface area and thickness measures, suggesting a particular involvement in hemispheric asymmetry rather than bilateral measures.

-Any single-sample GWAS should provide some measure of external validation of their findings in another sample (i.e. the reproducibility of loci or explained variance by a polygenic score etc.) since the use of only UK Biobank data is prone to overfitting.

Authors: Please see above where we have addressed this point in a response to the Editor (we added a new analysis in an independent sample, and drew attention to various other aspects that help to validate the results).

-For transparency and to allow other groups to make optimal use of the results, do the authors plan to make the GWAS summary statistics publicly available?

Authors: We have noted in the revised Data Availability Statement that the GWAS

statistics are made available online within the GWAS catalog https://www.ebi.ac.uk/gwas/ (this database publishes the statistics shortly after formal journal publication of the corresponding paper).

Reviewer #2:
Remarks to the Author:
The authors conducted a genome-wide association study (GWAS) of left-right asymmetry of various brain regions. The subjects were 32,256 participants in the UK Biobank. Despite the relatively small sample size by GWAS standards and the low GCTA heritabilities of the asymmetries, the authors were able to detect 21 genome-wide significant loci associated with different asymmetries. They employed MetaPhat, a new GWAS tool that implements canonical correlation analysis and uses a stepwise procedure to identify the components of the multivariate phenotype contributing to a significant canonical correlation. Using the enrichment tool MAGMA, they found that genes with low association p-value SNPs were unusually likely to be implicated in microtubule function. Despite what might have seemed a low statistical power to detect such enrichment, this finding seems statistically secure and is thus quite impressive and intriguing.
Some readers may be skeptical of the canonical correlation analysis. This may seem like a way to get something out of nothing. Without studying the specific method more closely, I think it is warranted to be skeptical of whether a significant multivariate hit can be followed up to determine the contributing components with an overwhelming degree of confidence. But in most of the downstream analyses, only the multivariate results are used, and I think the resulting inferences can be trusted. Readers can inspect Supplementary Table 11 to see the univariate associations that seem to be driving each multivariate hit, and this provides some transparency.
I recommend the publication of this paper, upon a few minor revisions and clarifications.

Authors: Thank you very much for these observations. As noted above in response to Reviewer 1, we have added more explanation for the multivariate approach, which has advantages in terms of data reduction and statistical power in the context of 42 regional asymmetry measures with relatively low but significant heritabilities. Indeed the univariate associations with each of the 'central' traits behind the multivariate associations can be found in the Supplementary Table 11, which are helpful to understand how each SNP relates to a subset of regional asymmetries.

p. 5, lines 103-104: "Seventy-two of the 112 genes have been reported to associate with educational attainment"
The cited paper used two different gene-prioritization tools, DEPICT and MAGMA (the one used by these authors). The DEPICT genes were nearly a subset of the MAGMA genes. Nevertheless, it will helpful to state which of the two lists of prioritized genes in the cited paper

were checked for overlap with the 112 asymmetry genes.

Authors: We used the MAGMA list from that paper because we used MAGMA for our own study. We have clarified this in the header of Supplementary Table 14. Please note that a preprint appeared in recent weeks https://www.biorxiv.org/content/10.1101/2020.08.20.260224v1 which found a problem with the 'SNP-wise mean model' implemented in MAGMA. The authors of MAGMA quickly fixed the problem, and we have updated all of our gene-based association testing and downstream analysis accordingly for this revision. In the updated results, there were 57 significant genes at Bonferroni-corrected p<0.05. Forty-three of the 57 genes have been reported to associate with educational attainment.

pp. 13-14, lines 328-334: There needs to be clarification of what qualifies a gene to "belong" to a particular stage. I.e., what makes a gene an "early prenatal" gene or a "late childhood" gene? Right now the authors say "differential mRNA expression (significantly higher gene expression)," but this is too vague.

Authors: Based on the gene-based association p-values for all 20,146 genes genome-wide, we used MAGMA (default settings as implemented in FUMA) to examine whether lower gene-based P values tended to be found for genes showing relatively higher expression in BrainSpan gene expression data from any particular ages compared to all other ages, separately for 29 different age groups ranging from 8 postconceptional weeks to 40 years old, and 11 defined developmental stages from early prenatal to middle adulthood. We corrected for multiple testing through a FDR of 0.05 (separately for the two analyses). Thus this is a quantitative analysis and does not require assigning genes categorically as e.g. 'early prenatal' etc., rather it just reflects that some genes tend to be expressed relatively more highly at some developmental stages.

We have now stated more clearly how this analysis works in the "Developmental stage analysis" section of Methods (page 19):
"Using the gene-based association p-values for all 20,146 genes genome-wide, we used MAGMA (default settings as implemented in FUMA) to examine whether lower gene-based p values tended to be found for genes showing relatively higher expression in BrainSpan[69] gene expression data from any particular ages compared to all other ages, separately for 29 different age groups ranging from 8 postconceptional weeks to 40 years old, and 11 defined developmental stages from early prenatal to middle adulthood. We corrected for multiple testing through a FDR of 0.05 (separately for the two analyses)."

Supplementary Figure 10: I suggest removing this figure. I don't think it is referred to in the main text. (The supplementary information refers to a "Supplementary Figure 14," but there are only

10 supplementary figures.) I think this kind of annotation is only informative if the SNPs have a good chance of being causal. Here, annotation of lead SNPs and all SNPs in LD r^2 > 0.6 is too inclusive.

Authors: We have removed Supplementary Figure 10 as we agree it was not helpful. We used the 0.6 threshold because it is the default in FUMA, and there is merit in sticking to default parameters to avoid making arbitrary decisions for SNP annotations and gene mapping. FUMA uses the same parameter also for clumping the associated SNPs into distinct genomic loci, so that altering this single parameter would affect both the inclusivity of candidate SNP labelling and the process of clumping. We therefore prefer to leave this at the default setting 0.6, with respect to sensible clumping. The issue does not affect our conclusions, as all of the notable functional annotations referred to in the text for the top loci remain if the threshold is increased to 0.8.

Decision Letter, first revision:

7th December 2020

*Please ensure you delete the link to your author homepage in this e-mail if you wish to forward it to your co-authors.

Dear Dr Francks,

Thank you once again for submitting your revised manuscript, entitled "The genetic architecture of structural left-right asymmetry of the human brain," and for your patience during the re-review process.

Your manuscript has now been evaluated by Reviewer 2, who also indicated that they are satisfied with your responses to Reviewer 1's comments, and in the light of their advice I am delighted to say that we can in principle offer to publish it. First, however, we would like you to revise your paper to ensure that it complies with our Guide to Authors at http://www.nature.com/nathumbehav/info/gta.

One of the main reasons for delays in formal acceptance is failure to fully comply with editorial policies and formatting requirements. To assist you with finalizing your manuscript for publication, I attach a checklist that lists all of our editorial policies and formatting requirements. I also attach a template document, which exemplifies our policies and formatting requirements.

Please attend to *every item* in the checklist and upload a copy of the completed checklist with your submission. I have highlighted in the checklist items that require your attention. I also mention here a few points that are frequently missed and can cause delays:

1) Ensure that all corresponding authors have linked their ORCID to their account on our online manuscript handling system. This is very frequently missed and invariably causes delays in formal

acceptance.

2) Ensure that you provide all of the materials requested in the attached checklist and below with your final submission. Please note that the Licence to Publish needs to be hand-signed.

3) Please include a sentence at the end of your Abstract noting that the findings that you report will need to be replicated in independent samples in the future.

Nature Human Behaviour offers a transparent peer review option for new original research manuscripts submitted from 1st December 2019. We encourage increased transparency in peer review by publishing the reviewer comments, author rebuttal letters and editorial decision letters if the authors agree. Such peer review material is made available as a supplementary peer review file. Please state in the cover letter ‘I wish to participate in transparent peer review’ if you want to opt in, or ‘I do not wish to participate in transparent peer review’ if you don’t. Failure to state your preference will result in delays in accepting your manuscript for publication.
Please note: we allow redactions to authors’ rebuttal and reviewer comments in the interest of confidentiality. If you are concerned about the release of confidential data, please let us know specifically what information you would like to have removed. Please note that we cannot incorporate redactions for any other reasons. Reviewer names will be published in the peer review files if the reviewer signed the comments to authors, or if reviewers explicitly agree to release their name. For more information, please refer to our FAQ page.

We hope to hear from you within 10 days; please let us know if the revision process is likely to take longer.

To submit your revised manuscript, you will need to provide the following:
• Cover letter
• Point-by-point response to the reviewers (if applicable)
• Manuscript text (not including the figures) in .docx or .tex format
• Individual figure files (one figure per file)
• Extended Data & Supplementary Information, as instructed
• Reporting summary
• Editorial policy checklist
• License to publish
• Third-party rights table (if applicable)
• Suggestions for cover illustrations (if desired)

Consortia authorship:
For papers containing one or more consortia, all members of the consortium who contributed to the paper must be listed in the paper (i.e., print/online PDF). If necessary, individual authors can be listed in both the main author list and as a member of a consortium listed at the end of the paper. When submitting your revised manuscript via the online submission system, the consortium name should be entered as an author, together with the contact details of a nominated consortium representative. See https://www.nature.com/authors/policies/authorship.html for our authorship policy and https://www.nature.com/documents/nr-consortia-formatting.pdf for further consortia formatting guidelines, which should be adhered to prior to acceptance.

Reviewer Recognition:
In recognition of the time and expertise our reviewers provide to Nature Human Behaviour's editorial process, we would like to formally acknowledge their contribution to the external peer review of your manuscript entitled "The genetic architecture of structural left-right asymmetry of the human brain". For those reviewers who give their assent, we will be publishing their names alongside the published article.

Please use the following link for uploading these materials:
[REDACTED]

If you have any further questions, please feel free to contact me.

With best regards,

Charlotte Payne
Editor
Nature Human Behaviour

Reviewer #2:
Remarks to the Author:
The authors have satisfactorily addressed the comments in the first round of review. I recommend the publication of this paper.

Final Decision Letter:

Dear Dr Francks,

We are pleased to inform you that your Article "The genetic architecture of structural left-right asymmetry of the human brain", has now been accepted for publication in Nature Human Behaviour.

Before your manuscript is typeset, we will edit the text to ensure it is intelligible to our wide readership and conforms to house style. We look particularly carefully at the titles of all papers to ensure that they are relatively brief and understandable.

Once your manuscript is typeset and you have completed the appropriate grant of rights, you will receive a link to your electronic proof via email with a request to make any corrections within 48 hours. If, when you receive your proof, you cannot meet this deadline, please inform us at rjsproduction@springernature.com immediately. Once your paper has been scheduled for online publication, the Nature press office will be in touch to confirm the details.

Acceptance of your manuscript is conditional on all authors' agreement with our publication policies

(see http://www.nature.com/nathumbehav/info/gta). In particular your manuscript must not be published elsewhere and there must be no announcement of the work to any media outlet until the publication date (the day on which it is uploaded onto our web site).

Nature Human Behaviour is a Transformative journal and offers an immediate open access option through payment of an article-processing charge (APC) for papers submitted after 1 January, 2021. In the event that authors choose to publish under the subscription model, Nature Research allows authors to self-archive the accepted manuscript (the version post-peer review, but prior to copy-editing and typesetting) on their own personal website and/or in an institutional or funder repository where it can be made publicly accessible 6 months after first publication, in accordance with our self-archiving policy. Please review our self-archviing policy for more information.

Several funders require deposition the accepted manuscript (AM) to PubMed Central or Europe PubMed Central. To enable compliance with these requirements, Nature Research therefore offers a free manuscript deposition service for original research papers supported by a number of PMC/EPMC participating funders. If you do not choose to publish immediate open access, we can deposit the accepted manuscript in PMC/Europe PMC on your behalf, if you authorise us to do so.

If you have posted a preprint on any preprint server, please ensure that the preprint details are updated with a publication reference, including the DOI and a URL to the published version of the article on the journal website.

An online order form for reprints of your paper is available at https://www.nature.com/reprints/author-reprints.html. All co-authors, authors' institutions and authors' funding agencies can order reprints using the form appropriate to their geographical region.

We welcome the submission of potential cover material (including a short caption of around 40 words) related to your manuscript; suggestions should be sent to Nature Human Behaviour as electronic files (the image should be 300 dpi at 210 x 297 mm in either TIFF or JPEG format). Please note that such pictures should be selected more for their aesthetic appeal than for their scientific content, and that colour images work better than black and white or grayscale images. Please do not try to design a cover with the Nature Human Behaviour logo etc., and please do not submit composites of images related to your work. I am sure you will understand that we cannot make any promise as to whether any of your suggestions might be selected for the cover of the journal.

You can now use a single sign-on for all your accounts, view the status of all your manuscript submissions and reviews, access usage statistics for your published articles and download a record of your refereeing activity for the Nature journals.

To assist our authors in disseminating their research to the broader community, our SharedIt initiative provides you with a unique shareable link that will allow anyone (with or without a subscription) to read the published article. Recipients of the link with a subscription will also be able to download and print the PDF.

As soon as your article is published, you will receive an automated email with your shareable link.

In approximately 10 business days you will receive an email with a link to choose the appropriate publishing options for your paper and our Author Services team will be in touch regarding any additional information that may be required.

You will not receive your proofs until the publishing agreement has been received through our system.

If you have any questions about our publishing options, costs, Open Access requirements, or our legal forms, please contact ASJournals@springernature.com

We look forward to publishing your paper.

With best regards,

Charlotte Payne
Editor
Nature Human Behaviour
